# Resilin matrix distribution, variability and function in *Drosophila*

**DOI:** 10.1186/s12915-020-00902-4

**Published:** 2020-12-14

**Authors:** Steven Lerch, Renata Zuber, Nicole Gehring, Yiwen Wang, Barbara Eckel, Klaus-Dieter Klass, Fritz-Olaf Lehmann, Bernard Moussian

**Affiliations:** 1 Applied Zoology, Technical University of Dresden, Dresden, Germany; 2 Animal Genetics, Interfaculty Institute of Cell Biology, University of Tübingen, Tübingen, Germany; 3 Senckenberg Natural History Collections, Dresden, Germany; 4 Animal Physiology, University of Rostock, Rostock, Germany; 5 CNRS, Inserm Institute of Biology Valrose, Université Côte d’Azur, Nice, France

**Keywords:** Resilin, Cuticle, Extracellular matrix, Drosophila, Flight

## Abstract

**Background:**

Elasticity prevents fatigue of tissues that are extensively and repeatedly deformed. Resilin is a resilient and elastic extracellular protein matrix in joints and hinges of insects. For its mechanical properties, Resilin is extensively analysed and applied in biomaterial and biomedical sciences. However, there is only indirect evidence for Resilin distribution and function in an insect. Commonly, the presence of dityrosines that covalently link Resilin protein monomers (Pro-Resilin), which are responsible for its mechanical properties and fluoresce upon UV excitation, has been considered to reflect Resilin incidence.

**Results:**

Using a GFP-tagged Resilin version, we directly identify Resilin in pliable regions of the *Drosophila* body, some of which were not described before. Interestingly, the amounts of dityrosines are not proportional to the amounts of Resilin in different areas of the fly body, arguing that the mechanical properties of Resilin matrices vary according to their need. For a functional analysis of Resilin matrices, applying the RNA interference and Crispr/Cas9 techniques, we generated flies with reduced or eliminated Resilin function, respectively. We find that these flies are flightless but capable of locomotion and viable suggesting that other proteins may partially compensate for Resilin function. Indeed, localizations of the potentially elastic protein Cpr56F and Resilin occasionally coincide.

**Conclusions:**

Thus, Resilin-matrices are composite in the way that varying amounts of different elastic proteins and dityrosinylation define material properties. Understanding the biology of Resilin will have an impact on Resilin-based biomaterial and biomedical sciences.

## Background

Elasticity and resilience are essential for the integrity of tissues that function through repeated and extensive deformation. In vertebrates, for instance, the elastic extracellular polymeric protein Elastin in the lung and blood vessels ensures their functionality after numerous times of use [1, 2]. The insect exoskeleton (cuticle) is subdivided into rigid and pliable regions that in concert allow various types of movement like running, jumping, crawling, biting and flight. An essential element of the pliable regions is an elastic extracellular protein-matrix called Resilin [3, 4]. Resilin usually resides in joints [5], wing articulations [6] and adhesive structures at the end of limbs [7]. The mechanical properties of Resilin have been studied extensively in vivo and in vitro for biomedical and biomimetic purposes. Despite rapid progress in this respect, the composition of the Resilin matrix is yet unexplored. Conceptually, the Resilin matrix consists of Resilin monomers (Pro-Resilin) that are cross-linked via di- and trityrosine bonds [8–10]. Upon excitation with UV light, di- and trityrosines emit blue light, a property that has been used for indirect Resilin detection in various insects [11, 12]. Generally, Pro-Resilin has a type 2 Rebers and Riddiford consensus chitin-binding motif (R&R-2) suggesting that association with the polysaccharide chitin is needed for Resilin function [13–15]. In the fruit fly *Drosophila melanogaster*, besides the full-length Pro-Resilin isoform with the R&R-2 domain (620 aa), an isoform lacking this domain (575 aa) is present through alternative splicing. Outside the R&R-2 motif, Pro-Resilin sequences in different insects vary considerably. In principle, these sequences are characterised by stretches of non-conserved repetitive blocks that account for the elasticity of Pro-Resilins [10, 16–18].

Presence of Resilin in different body parts argues that matrix composition may vary to accommodate the respective mechanical need. For instance, in adhesive tarsal setae, a fluorescence gradient implying a Pro-Resilin concentration gradient has been reported [18], while in joints and jump devices, a massive extracellular Resilin matrix prevails [5, 19]. This variety suggests that along with Pro-Resilin, other proteins may be incorporated in the Resilin matrix to modify the mechanical properties according to its role in movement and adhesion. Detailed knowledge about the constitution and configuration of different Resilin matrices would allow understanding of how matrix properties are modulated according to specific needs. To address the function of Resilin matrices in an insect, we chose to first determine directly the tissue distribution of Pro-Resilin in the cuticle of adult *D*. *melanogaster* fruit flies and second to behaviourally characterise flies without Pro-Resilin through genetic manipulation.

## Results

### Resilin-GFP distribution in the fly body

**Fig. 1:**
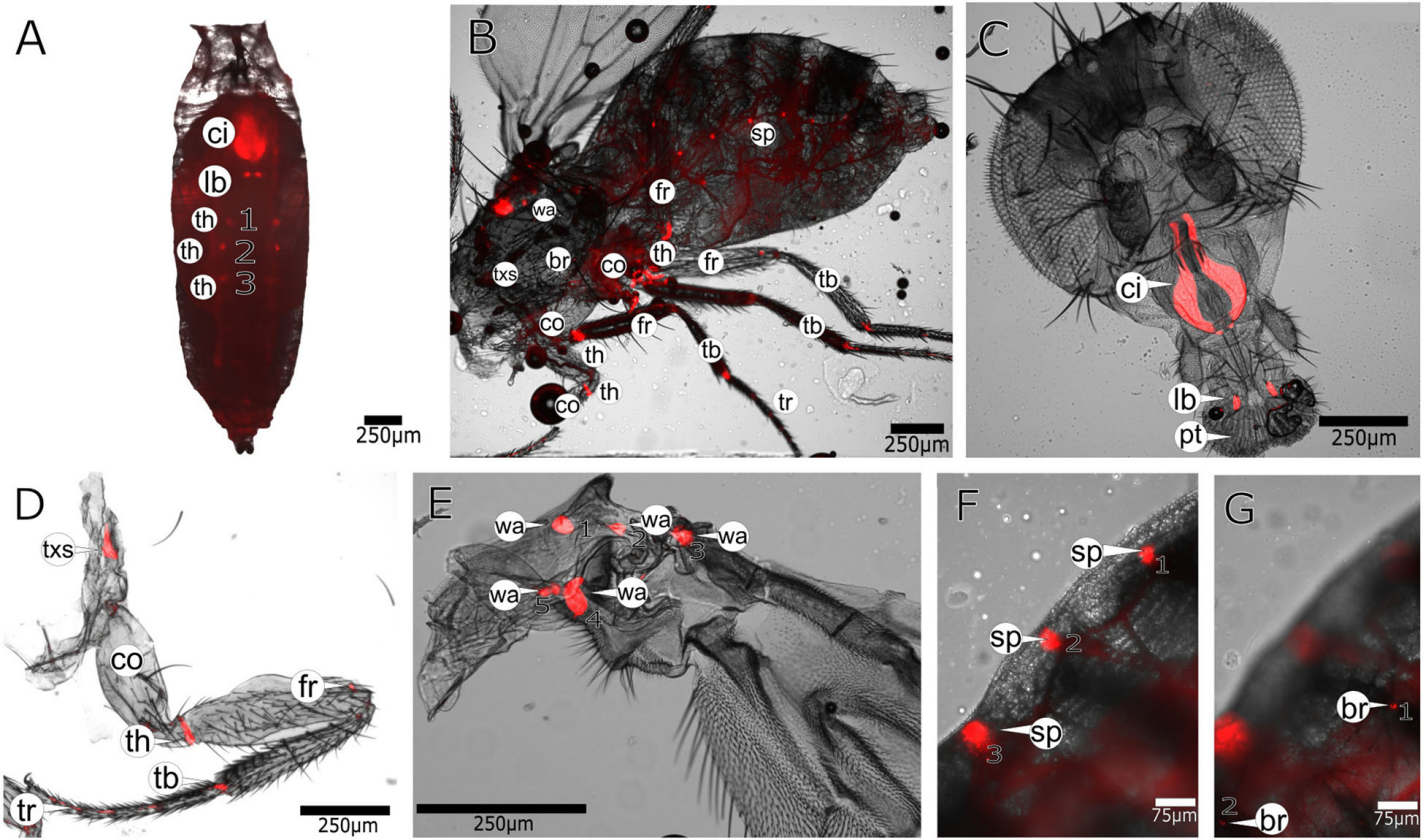
Resilin localises to distinct regions of the fly body. **a** Pro-Resilin-GFP (red) is detected in the head region of the pupa. **b** In the adult fly, spots are found at the leg joints, at the wing articulation and on the flanks of the abdomen. **c** In the head, Pro-Resilin-GFP marks large patches around the cibarium and the labellum. **d**, **e** By higher magnification, several small Pro-Resilin-GFP patches are detected along the length of the leg, close to the joints and at the wing articulations. Details of the Pro-Resilin-GFP signal in the leg are shown in Additional file 3: Figure S3. **f** Small spots are visible at the tracheal endings. **g** Pro-Resilin-GFP is also detected at the hair bases. The GFP signal was merged with the bright-field image in **a**–**g**. Images were recorded on a Leica DMi8 microscope. Details of the microscope settings are described in the “Methods” section. Labelling: br, bristle; ci, cibarium; co, coxa; fr, femur; lb, labellum; sp, spiracle; tb, tibia; th, trochanter; tr, tarsus; txs, thorax, exact location of signal unclear; wa, wing articulation

**Fig. 2:**
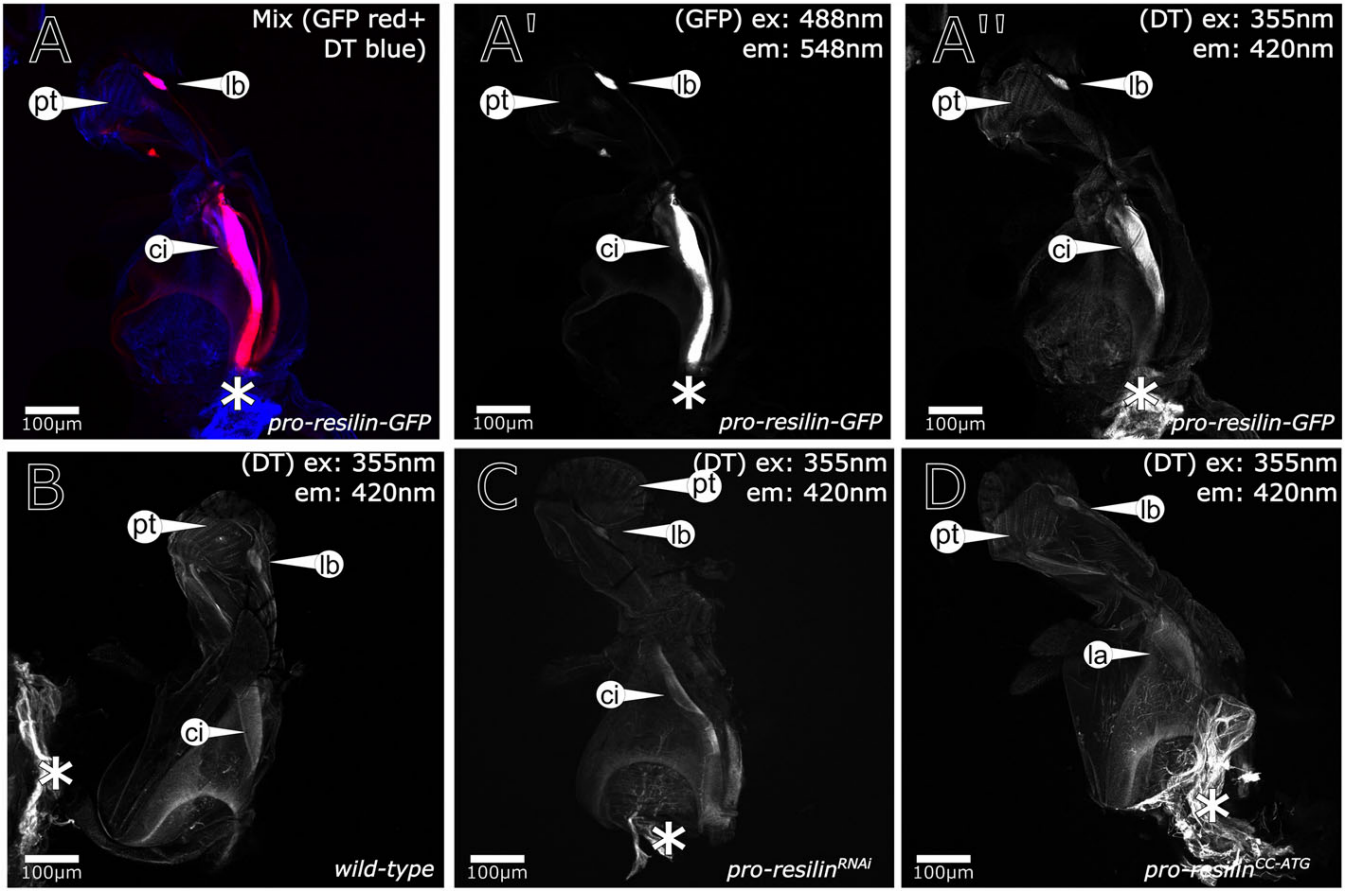
Pro-Resilin-GFP is expressed in the proboscis. **A**, **A′** The labellum and the cibarium (http://flybase.org/cgi-bin/cvreport.pl?id=FBbt:00004526) contain large amounts of Pro-Resilin-GFP. **A**, **A″** These areas are marked by DT as in non-transgenic wild-type flies (**B**). **C**, **D** In *pro-resilin*^RNAi^ and *pro-resilin*^*cc-ATG*^ flies the DT signal persists. Reduction of the DT signal is detectable by the Fiji software. Occasionally, the pseudotrachea (pt) (http://flybase.org/reports/FBim0000834) do not contain Pro-Resilin-GFP, but display a weak auto-fluorescence. We consider this as background signal. The asterisk (*) marks auto-fluorescence of internal tissues after dissection. Images were generated with a Zeiss LSM880 confocal microscope. The excitation (ex) and emission (em) wavelengths are indicated in the images. Those shown in **A**–**A‴** were obtained by the normal confocal mode, while those shown in **B**–**D** were produced by the fast airyscan mode. Details of the respective settings are described in the “Methods” section. Labelling: ci, cibarium; lb, labellum; pt, pseudotrachea

**Fig. 3:**
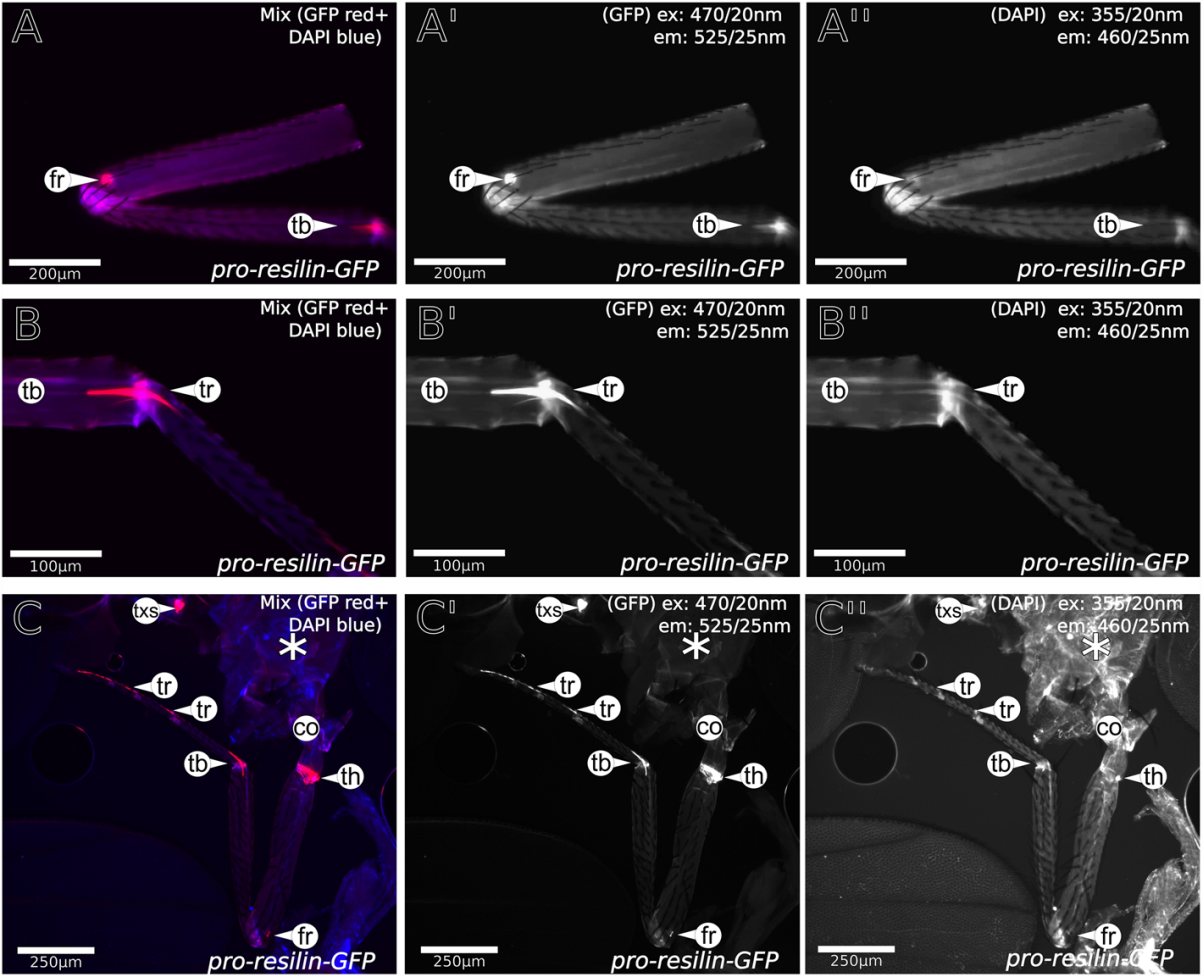
In legs, some Pro-Resilin-GFP signal does not coincide with a DT signal. **A**–**A″** At the femur-to-tibia transition, we detect a Pro-Resilin-GFP patch, that does not coincide with a DT signal. **B**–**B″** In the joint between the tibia and the tarsus, there is a star-like area of Pro-Resilin-GFP signal. This patch is not covered completely with a DT signal. **C**–**C″** The tarsi show an array of Pro-Resilin-GFP signal. The DT signal does not overlap perfectly with the Pro-Resilin-GFP signal. The asterisk (*) marks auto-fluorescence of internal tissues after dissection. A Leica DMi8 microscope was used for imaging. The excitation (ex) and emission (em) wavelengths are indicated in the images. Details of the microscope settings are described in the “Methods” section. Labelling: fr, femur; th, trochanter; tb, tibia; tr, tarsus; txs, thorax, exact location of signal unclear

**Fig. 4:**
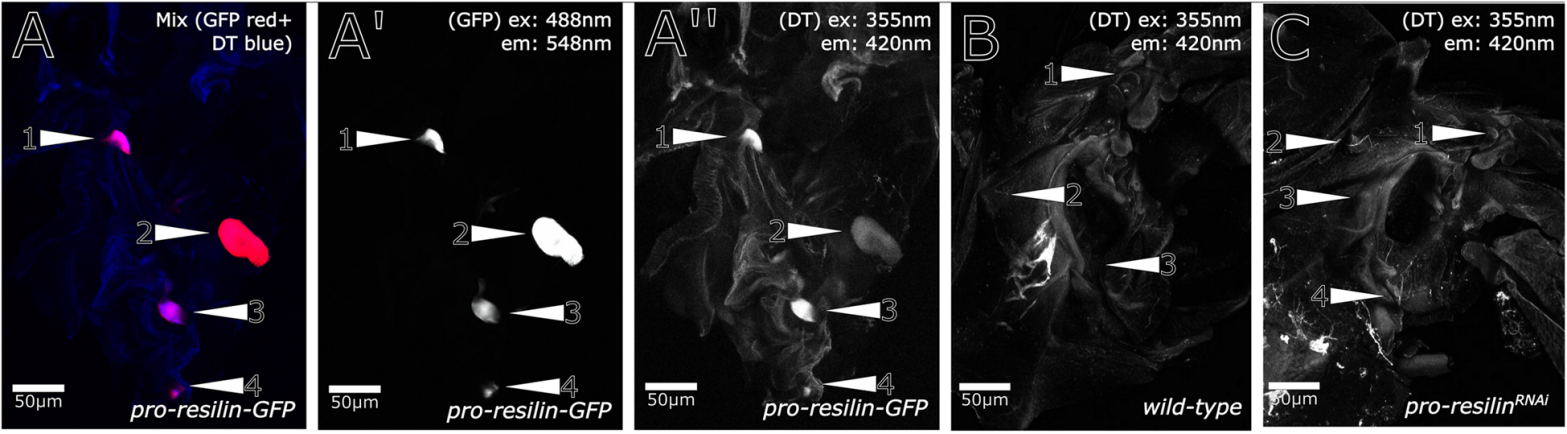
Several patches of Pro-Resilin-GFP signal characterise the wing articulation. As shown in Fig. 1, Pro-Resilin-GFP marks five patches in the wing articulation. **A**–**A″** By confocal microscopy, occasionally, one or two of these patches are out of focus and are therefore not visible. It is, however, evident that the DT signal intensity varies independently of the signal intensity of GFP. **B** The DT signals are also present in wild-type non-transgenic flies. **C** DT signal intensity is obviously reduced in *pro-resilin*^*RNAi*^ flies. Patches are numbered in each image. The asterisk (*) marks auto-fluorescence of internal tissues after dissection. Images were generated on a Zeiss LSM880 confocal microscope. The excitation (ex) and emission (em) wavelengths are indicated in the images. They were obtained by the normal confocal mode. Details of the respective settings are described in the “Methods” section

**Fig. 5:**
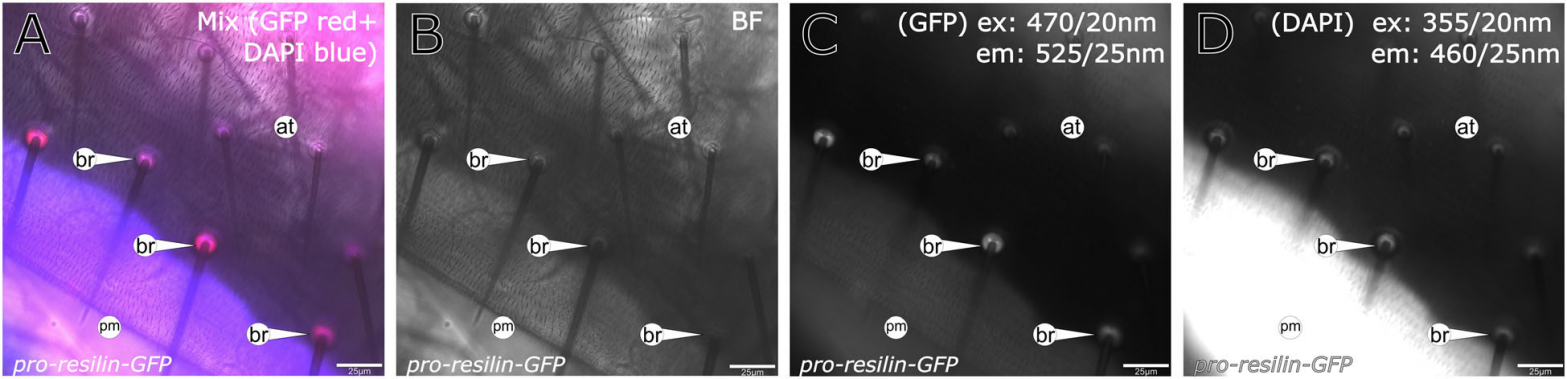
Pro-Resilin-GFP is present in the bristle socket. Pro-Resilin-GFP (red signal, **a** and **c**) marks the socket of bristles (br) in abdominal tergites (at, bright-filed image shown in **b**). Autofluorescence (blue signal, **a** and **d**) excited by a 355 nm light source, possibly corresponding to the presence of DT, is also detected in the bristle sockets. The pleural membrane (pm) displays a strong autofluorescence under these conditions; therefore, presence of DT is uncertain. Signals were detected on a Leica DMi8 microscope. The excitation (ex) and emission (em) wavelengths are indicated in the images. Details of the microscope settings are described in the “Methods” section. Labelling: at, abdomnal tergite; br, bristle; pm, pleural membrane


In order to visualise Resilin in *D*. *melanogaster*, we used transgenic flies expressing a C-terminally GFP-tagged Pro-Resilin (Pro-Resilin-GFP) under the control of the endogenous promoter (Additional file 1: Figure S1). Pro-Resilin-GFP is, in contrast to recent reports [20], not expressed during embryogenesis and larval development. This was confirmed by quantitative real-time PCR (qPCR; Additional file 2: Figure S2). During pupal development, patches of GFP signal were detected in the wing articulation and near the leg joints, probably basal parts of tendons (Fig. 1a, Additional file 16: Movie S1). In addition, there was a large area of GFP expression in the proboscis (Fig. 1a). The pupal expression pattern persisted in adult flies (Fig. 1b–g). A detailed analysis of Pro-Resilin-GFP in the proboscis, the leg and the wing articulation is shown in Figs. 2, 3 and 4, respectively. Close inspection of bristles revealed that the bases of only those on the posterior segments of the abdomen contained Pro-Resilin-GFP (Fig. 1g, Fig. 5, Additional file 4: Figure S4). Moreover, the segmental openings of the tracheae (spiracles) at lateral position of the body were marked by Pro-Resilin-GFP (Fig. 1f, Additional file 4: Figure S4). In adult females, the GFP signal was detected in spermatheca ducts (Additional file 5: Figure S5). This localization pattern confirms largely previous predictions, assumptions and descriptions of Resilin occurrence in various insect species [21–23].

**Additional file 16: Movie S1.** Expression of Pro-Resilin-GFP in *D*. *melanogaster* during metamorphosis. This is a time-lapse of 15 min recorded by a Leica DMi8 microscope. GFP is shown in red.

### Resilin-GFP colocalises with dityrosine

Usually, histological identification of Resilin in insects relied indirectly on the presence of dityrosine bonds (DT) between Pro-Resilin monomers that confer auto-fluorescence when excited with UV light [7, 8, 24]. To test to what extent presence of Pro-Resilin-GFP coincides with DT formation, we scanned the cuticle of Pro-Resilin-GFP flies with a 355-nm laser beam that excites DT auto-fluorescence (Figs. 2, 3, 4, Additional file 4: Figure S4). The majority of the GFP patches coincided with DT auto-fluorescence. In few Pro-Resilin-GFP regions including a remarkable strong spot in the femur (Fig. 3A), parts of the tibia-femur joint (Fig. 3B) and the tarsi (Fig. 3C), the GFP signal was not accompanied by a DT signal.

Generally, in the whole *D*. *melanogaster* body, no convincing DT signal was detected that did not merge with a Pro-Resilin-GFP signal (see Figs. 2, 3, 4, 5 and Additional file 11: Figure S11). However, we are reluctant to exclude such a situation as we observed that occasionally the tips of the proboscis, for instance, that were devoid of any Pro-Resilin-GFP signal, displayed weak auto-fluorescence when excited with a light of 355 nm (Fig. 2). By trend, nevertheless, these findings suggest that commonly all DT regions in the fly body cuticle include Pro-Resilin-GFP. Some Pro-Resilin-GFP areas, however, did not show any DT signal suggesting that dityrosinylation of Pro-Resilin is not obligatory.

### Cross-linking of Resilin-GFP varies in different regions of the fly body

**Fig. 6:**
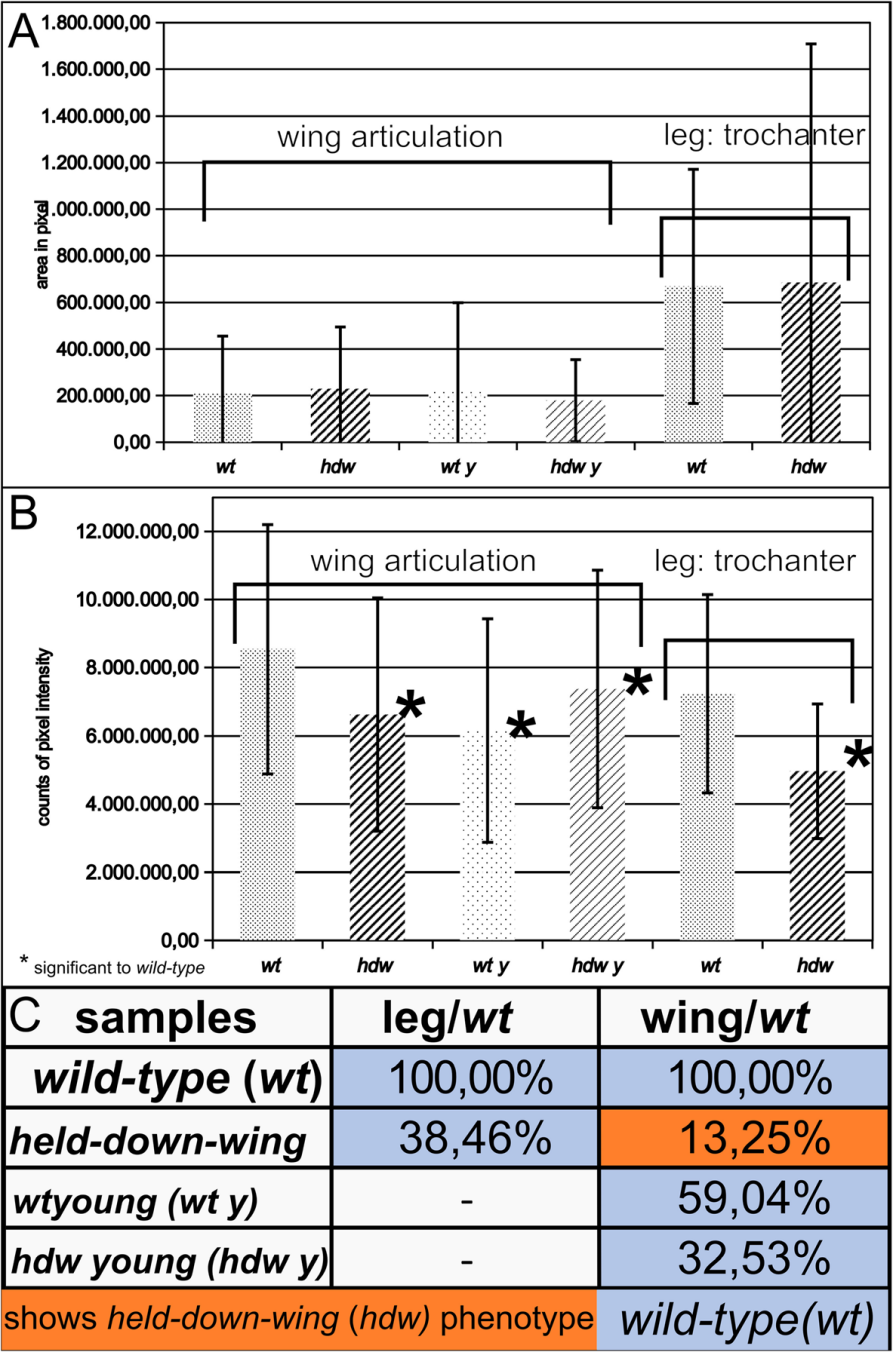
DT signal intensities are reduced in *pro-resilin*^*RNAi*^ flies. We measured and compared the DT signal intensities in wild-type and *pro-resilin*^*RNAi*^ wing articulations and trochanter following two statistical calculations. First, intensities were simply averaged and compared. In the second approach, we chose the intensity of 4 × 10^6^ counts/pixel as a threshold value to test how many samples passed this threshold (see the “Methods” section). **a** The areas of the wing articulations and the trochanter have similar sizes in wild-type and *pro-resilin*^*RNAi*^ flies. **b** In one approach, the DT intensities in both the wing articulations and the trochanter were significantly reduced in *pro-resilin*^*RNAi*^ flies compared to wild-type flies. **c** According to the other approach, 100% of wild-type wing articulations and trochanter had at least an intensity value of 4 × 10^6^ counts/pixel, while only about 13% and 38% of *pro-resilin*^*RNAi*^ wing articulation and trochanter samples, respectively, reached this value. This method also revealed that at least in the wing articulations, the DT signal accumulates during ageing. This accumulation is attenuated in *pro-resilin*^*RNAi*^ wing articulations. The sample sizes (*n*) were as follows: wild-type legs 64, *pro-resilin*^*RNAi*^ legs 62, wild-type (old and young) and *pro-resilin*^*RNAi*^ wing articulations 35

We observed that the ratio of Pro-Resilin-GFP and DT signals differed between the Resilin regions. For example, the intensities of the five Pro-Resilin-GFP spots in the wing articulation did not match the intensities of the DT signals at these positions (Fig. 4A, A″). These differences indicate that the degree of dityrosinylation is not proportional to the amounts of Pro-Resilin in Resilin matrices in the adult fly. Similarly, in the desert locust *Schistocerca gregaria* degree and distribution of Resilin cross-linking is regionalized [25]. Moreover, we observed that the DT signal accumulated over time in the adult fly (Fig. 6). To test whether presence of Pro-Resilin is sufficient to induce DT formation, we ectopically expressed a C-terminally Venus-tagged Pro-Resilin version [26] in the epidermis and monitored auto-fluorescence in Venus-positive tissues. No DT signal was detected in ectopic Pro-Resilin-Venus locations indicating that the tissues naturally expressing Pro-Resilin are at the same time competent in DT formation (data not shown).

### Pro-Resilin reduction or elimination is not lethal

**Fig. 7:**
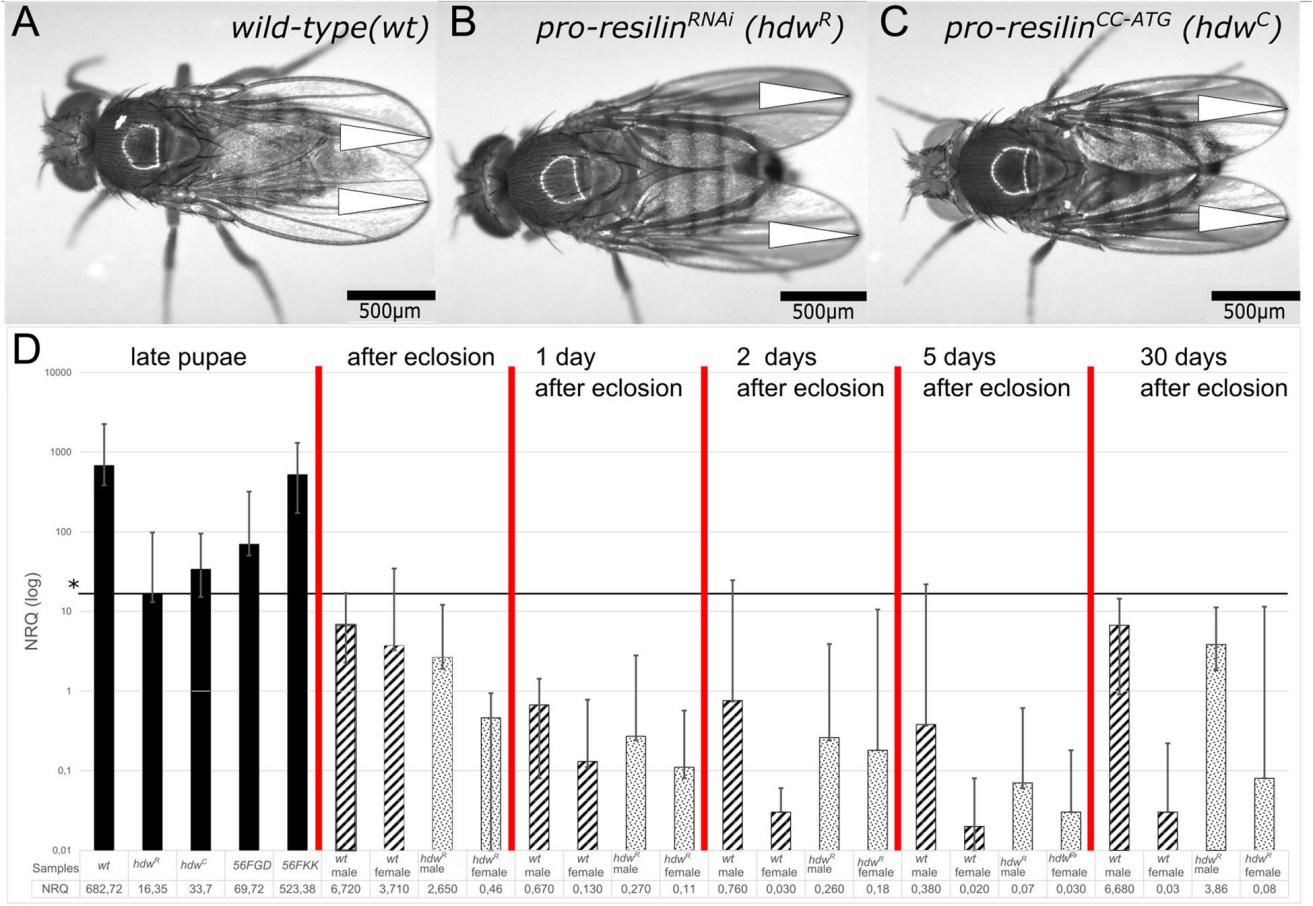
Reduction and elimination of *pro-resilin* cause a wing posture phenotype. **a** In immobile wild-type flies, wings are flat and arranged at the dorsum (triangles). **b**, **c** In flies with reduced (*pro-resilin*^*RNAi*^) or eliminated (*pro-resilin*^*CC*^) Pro-Resilin function, the wings are held down (triangles, hdw for held-down wing phenotype). This phenotype is nicely visible in the Additional files 19, 20, 21, 22, 23, 24: movies S4, S5, S6, S7, S8, S9. **d** In wild-type flies, *pro-resilin* is expressed throughout pupal development. Expression drops at eclosion and remains low during adult life after 1, 2, 5 and 30 days. Expression is several hundred times lower in *pro-resilin*^*RNAi*^ and *pro-resilin*^*CC*^ flies (non-overlapping confidence intervals, CI, indicate significant difference in expression level). Males and females have been analysed separately after eclosion. NRQ, normalised relative quantity. Images were generated with a Leica EZ4HD with an in-built camera


In order to study the biological importance of Resilin, we ubiquitously knocked down *pro-resilin* expression in flies by RNA interference (RNAi) using the UAS/Gal4 expression system. RNAi significantly reduced *pro-resilin* expression (Fig. 7). These flies (*pro-resilin*^*RNAi*^) were unable to fly (Additional files 17 and 18: Movies S2 and S3). This phenotype underlines that the Pro-Resilin-GFP-marked patches in the thorax are essential for flight. The morphology of the wing articulation in the thorax was slightly changed in these flies (Additional file 6: Figure S6). In addition, reduction of *pro-resilin* expression caused a wing posture failure within one day after eclosion (Fig. 7, Additional file 19: Movie S4). The held-down wing (*hdw*) phenotype suggests increased fatigue of the Pro-Resilin containing patches. Thus, both flapping and holding of the wing depend on Pro-Resilin. To analyse to what extent wing posture failure affects wing function other than for flight, we filmed *pro-resilin*^*RNAi*^ flies during courtship and mating. Wild-type males performed a courtship dance among others moving their wings (Additional file 20: Movie S5). In contrast, *pro-resilin*^*RNAi*^ males did not use their wings during courtship (Additional file 21: Movie S6). They nevertheless were able to mate with females and produce offspring. An interesting conclusion from these observations is that Resilin is not only needed for movement in the wing articulation but has also a static function in this structure.

**Additional file 17: Movie S2.** Flight by a wild-type fly. A female with is fixed to a metal support to film wing articulation mobility.


**Additional file 18: Movie S3.** Inability of flight by a female fly with a hdw phenotype. A female with *pro-resilin*-^*RNAi*^ induced reduction of Pro-Resilin is fixed to a metal support to film wing articulation mobility.


**Additional file 19: Movie S4.** Manifestation of the hdw phenotype. Two males are kept in a small vial preventing flight. The male in the lower position shows the hdw phenotype after some minutes.


**Additional file 20: Movie S5.** Wild-type *D*. *melanogaster* courtship behaviour. A male and a female were kept in a small vial and filmed during courtship using their wings. At the end, they mated.


**Additional file 21: Movie S6.** Courtship of hdw phenotype flies. A male and a female both with a hdw phenotype were kept in a small vial and filmed during courtship. They mated despite of courtship failure.

In order to test whether the phenotype of *pro-resilin*^*RNAi*^ flies is specific and not due to any off-target effect, we knocked down *pro-resilin* function by RNAi in flies expressing Pro-Resilin-GFP that has a 3′ region not recognized by the hairpin RNA. The resulting flies had a normal wing posture indicating that the *hdw* phenotype of *resilin*^*RNAi*^ flies is caused specifically by RNAi mediated *pro-resilin* knock-down (Additional file 7: Figure S7).

To further underline the specificity of the *pro-resilin* deficient *hdw* phenotype, we generated *pro-resilin* mutant flies using the Crispr/Cas9 technology. A guide RNA recognising the region around the start codon was injected in embryos of flies ubiquitously expressing *cas9*. Several flies were recovered that displayed the *hdw* phenotype. Sequencing of the *pro-resilin* gene in flies stably segregating *pro-resilin* mutations revealed various types of deletion mutations that lead to translation of aberrant and shortened Pro-Resilin proteins (Additional files 8 and 10: Figure S8 and S10). These mutations naturally affect both the short and the long Pro-Resilin isoforms. Homozygous *pro-resilin*^*cc-ATG*^ mutant flies showed the same phenotype as the *pro-resilin*^*RNAi*^ flies (Fig. 7). To scrutinise whether the Rebers and Riddiford (R&R) chitin-binding domain, which mediates chitin binding encoded by the second, alternative exon is needed for Pro-Resilin function, we mutated this exon using a respective guide RNA and obtained several *pro-resilin*^*cc-R&R*^ mutant fly lines. These flies, by consequence, have a normal, short Pro-Resilin isoform (without the R&R domain) and an isoform with a disrupted R&R domain. Mutations in this exon cause a phenotype indistinguishable from the phenotype caused by mutations around the start codon (Additional files 22, 23, 24: Movies S7, S8, S9). This result demonstrates that the chitin-binding domain is crucial for Pro-Resilin function. To further understand the relationship between Resilin and DT, we monitored the intensity of DT in flies with suppressed *pro-resilin* expression (Figs. 2 and 4; Additional file 11: Figure S11). The DT signal was markedly reduced in the wing articulation of these flies (Figs. 4 and 6). In the leg joints and the proboscis, reduction of DT signal was less pronounced (Figs. 2 and 6, Additional file 11: Figure S11). Thus, DT signal persists to a varying degree in regions with reduced Pro-Resilin.


**Additional file 22: Movie S7.** Wild-type flies move freely in a polystyrene multi-well plate with a lid. Their wings are kept on their backs.


**Additional file 23: Movie S8.** Flies homozygous for the *pro-resilin*^*CC-RR*^ allele move freely in a polystyrene multi-well plate with a lid. They keep their wings laterally (hdw phenotype). Of note, we also observe that *pro-resilin*^*CC-RR*^ flies are able to climb up the wall of the well suggesting that the tarsi are normal.


**Additional file 24: Movie S9.** Flies homozygous for the *pro-resilin*^*CC-ATG*^ allele move freely in a polystyrene multi-well plate with a lid. They keep their wings laterally (hdw phenotype). Of note, we also observe that *pro-resilin*^*CC-ATG*^ flies are able to climb up the wall of the well suggesting that the tarsi are normal.

In a series of assays, we studied the biological role of Pro-Resilin in the adult fly beyond flight and courtship (see above). To test whether Resilin in the tracheal openings is needed for the gate function of these structures, *pro-resilin*^*cc-ATG*^ flies were incubated with the dye Eosin Y in a penetration assay [27, 28]. Eosin Y did not enter the tracheae of wild-type or *pro-resilin* deficient flies (Additional file 12: Figure S12). This suggests that Resilin is not essential for the gate function at the tracheal openings. In feeding experiments, we sought to fathom whether Pro-Resilin has a function in the proboscis. In CAFE assays [29], we were unable to discern any difference in food uptake between *pro-resilin*^*cc-ATG*^ and wild-type control flies (*n* = 60 flies [6 replicates à 10 flies], *p* = 0.23–0.54, Student’s *t* test). A more sophisticated experiment supplying food with different densities is needed to further study this issue. To analyse the biological role of Resilin in the spermathecal ducts of females, we assessed fertility. For this purpose, we mated wild-type male flies with *pro-resilin* mutant females for 4 h (to avoid double mating). Fertility of these females (*n* = 31) was not reduced significantly (*p* = 0.26, Student’s *t* test) compared to wild-type females (*n* = 14). We conclude that under laboratory conditions the function of Resilin in female reproduction, i.e. spermathecal ducts is dispensable.

### DT is present in Pro-Resilin-less flies

Resilin-less flies are viable, and the Resilin-associated DT signal is not completely abolished in these flies (Fig. 6). Non-lethality may indicate that *pro-resilin* is not essential for survival at least in the laboratory or that a Resilin-like protein compensates for the reduction or elimination of Pro-Resilin function. A candidate for this redundant function is Cpr56F, a cuticular R&R-domain protein related to Resilin as proposed by Ardell and Andersen and Karouzou et al. [13, 30]. Cpr56F is smaller than Pro-Resilin (217 aa versus 575/620 aa, Additional files 9 and 10: Figure S9 and S10) and contains two repeats flanking the R&R domain with a total of 14 tyrosines. To analyse a possible redundancy between Pro-Resilin and Cpr56F by phenotype comparisons, we generated flies with reduced or eliminated Cpr56F function. First, we ubiquitously downregulated Cpr56F expression by RNAi. Downregulation of *cpr56F* expression is not lethal, and the respective flies do not display any obvious phenotype (Additional file 13: Figure S13). Next, we mutated the coding region of *cpr56F* applying the Crispr/Cas9 technique and analysed the respective mutant phenotype. We obtained several alleles that were all lethal before pupation and therefore not directly amenable to phenotypic analyses in the adult.

### Cpr56F may cooperate with Pro-Resilin in some Resilin regions

**Fig. 8:**
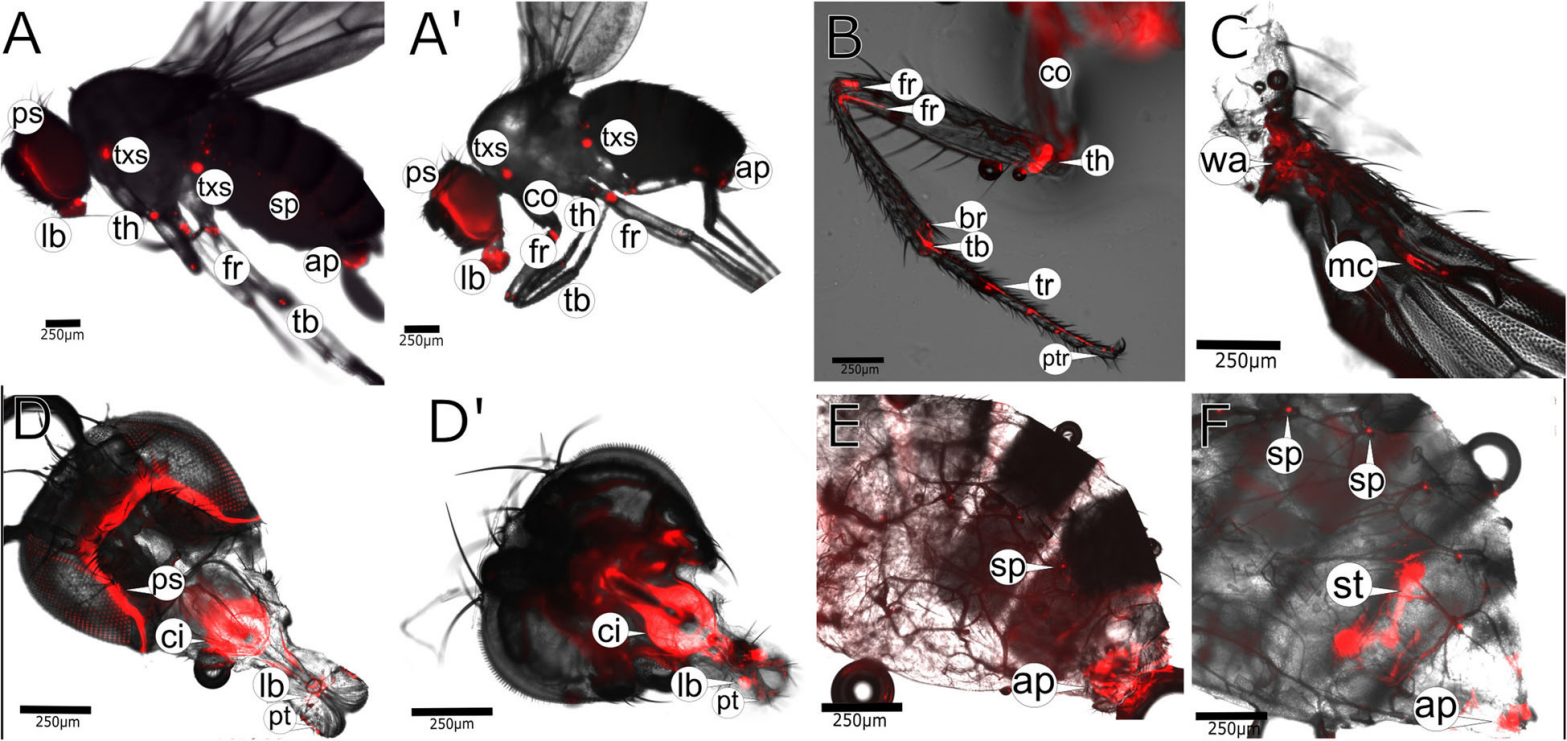
Cpr56F partially localises to those tissues that also express Pro-Resilin-GFP. **A** In the adult fly (female, A' male), Cpr56F-GFP is present as large or small patches in the head, the legs and the abdomen. **B** All leg joints are marked by Cpr56F-GFP (triangles). **C** Cpr56F-GFP is present in the wing basis (wa) and in the proximal rim of the marginal cell (mc). **D** The edge of the back head contains Cpr56F-GFP (ps). **D′** The cibarium and the labellum are framed by a strong GFP signal (ci, lb. and pt). **E** The male abdominal ending is marked by a patch of Cpr56F-GFP signal (ap). Also, their spiracles are marked by Cpr56F-GFP (sp). **F** Cpr56F-GFP localises to the spermathecae of females (st). Their tracheal endings also contain Cpr56F-GFP (sp). The end of the female abdomen shows a distinct Cpr56F-GFP signal (ap). The GFP signal was merged with the bright-field image in **A**–**F**. Images were recorded on a Leica DMi8 microscope. Details of the microscope settings are described in the “Methods” section. Labelling: Ap, anal plate; br, bristle; ci, cibarium; co, coxa; fr, femur; it, integument; lb, labellum; mc, marginal cell; ps, ptilinal suture; ptr, pretarsus; sp, spiracle; st, spermatheca; tb, tibia; th, trochanter; tr, tarsus; txs, thorax, exact location of signal unclear

Identical or similar spatial expression pattern may also be indicative of redundancy. We recorded the expression pattern of the chimeric Cpr56F:GFP protein that was expressed under the control of its endogenous promoter (Additional file 1: Figure S1). Expression of Cpr56F-GFP was first detected in larvae (Additional file 14: Figure S14). In adults, the Cpr56F-GFP signal was seen in leg joints (Fig. 8A, B), the head (including proboscis, Fig. 8A, D), the spermathecal duct (Fig. 8F), at the end of the abdomen (Fig. 8E, F) and the tracheal openings (Fig. 8E, F). Importantly, it was present at the wing hinge and at the proximal rim of the marginal cell of the wing blade but not in the wing articulation (Fig. 8C). Hence, some Cpr56F-GFP signal coincided with regions of Pro- Resilin-GFP expression, but some did not. Interestingly, none of the regions that showed a Cpr56F-GFP but not a Pro-Resilin-GFP signal (i.e. wing hinge and wing blade) seemed to unambiguously contain DT (see Figs. 2, 4, and Additional file 11: Figure S11). Regarding the wing blade, in a number of insects, which are much bigger than *D*. *melanogaster* including honeybees and dragonflies, DT was found at positions critical for flight [31]. To test whether a possible DT signal at the proximal rim of the marginal cell may depend on the size of the insect, we examined the wing blade of the bigger *Drosophila hydei* (Additional file 15: Figure S15). In the wing blade of this species, a distinct DT signal at this position was observed. We conclude that a possible DT signal in *D*. *melanogaster* is too weak for unambiguous detection.

Together, we postulate that Pro-Resilin and Cpr56F act together in some cuticle regions under continuous mechanical stress, while in some other regions, Pro-Resilin and Cpr56F act alone or with another yet unidentified Resilin-like protein.

## Discussion

### Pro-Resilin localises to various body cuticle regions and organs in the adult fly

Pro-Resilin is exclusively expressed during metamorphosis. By qPCR, confirming our Pro-Resilin-GFP data and modEncode and others expression analyses [10, 32], we find that *pro-resilin* is not expressed during embryogenesis and larval stages of *D*. *melanogaster*. This finding contradicts previous localization data using a Pro-Resilin-specific antibody called Rec-1 [20]. Rec-1 is reported to recognise an epitope already in the epidermis of the embryo. This antibody was also used to detect “Resilin” in the jumping plant bug *Philaenus spumarius* [33]. Based on our histological data, we propose to be careful in interpreting these data. In contrast to Pro-Resilin, Cpr56F is expressed in larvae, namely in the foregut. Possibly, the constant use of the foregut in swallowing food is facilitated by the use of Cpr56F-mediated elasticity of the foregut cuticle. In any case, the larval body cuticle is devoid of Pro-Resilin. Thus, larval body cuticle elasticity and pliability rely on other cuticle matrix components. A dityrosine layer in-between the epi- and the procuticle that we discovered recently [26] may bear these properties.

In the adult body, Resilin (Pro-Resilin and/or Cpr56F) is detected in the tracheal endings, the spermatheca ducts, at the wing articulations, at or near to limb joints and the proboscis. Those body parts used for locomotion and movement (wing articulations, limb joints) have already been described extensively to require Resilin in other insect species [7, 11]. In *D*. *melanogaster*, these parts are comparably small and difficult to denominate. We think that the five Pro-Resilin patches in the thorax may represent attachment sites of the steering muscles [34].

Besides the information on Resilin distribution in the fly body, it is interesting to note where Resilin is absent. In many insect species including honeybees and dragonflies, DT, i.e. Resilin has been reported to occur at the margins of wing veins [35–37]. In *D*. *melanogaster*, we did not detect any Pro-Resilin or DT signal associated with wing veins. Hence, flight mechanics may correlate with wing size and are different in different species.

Resilin patches in and adjacent to the joints are relatively smaller than those regions described in the legs of jumping insects where they serve energy storage and release (e.g. [38]). Like in wing articulations, we assume that Resilin patches near the joints represent attachment sites for muscles.

The spiracles at the tracheal endings control gas exchange and water loss by respiration [39]. A repeated opening and closing of the valves that are of cuticular origin apparently necessitates the resilience of spiracle cuticle matrix containing Pro-Resilin and Cpr56F. Before this work, Resilin had not been reported to participate at spiracle formation and function.

The largest body area that harbours Resilin is the proboscis. In lepidopteran species, recoiling of the proboscis seems to require Resilin in the dorsal half of the proboscis wall [22]. In the fly proboscis, Resilin may have two functions: (1) in analogy to the situation in lepidopterans, it is needed for proboscis retraction, and (2) it sustains repeated food pumping [40].

Taken together, in the *D*. *melanogaster* adult body, in conjunction with non-Resilin containing cuticle parts, Resilin contributes small matrices to skeletal structures, organs and tissues that are subject to repeated movement, flexion or torsion. Its function in these structures remains to be studied in biomechanical experiments.

### The Pro-Resilin-DT ratio is not proportional

Pro-Resilin is composed of two types of repeat motifs, named A and B [13] that flank the chitin-binding domain (Additional file 8: Figure S8). Outside the chitin-binding domain, there are 36 tyrosines (there is no tyrosine in the signal peptide). Cpr56F has two repeats and 14 tyrosines outside of the chitin-binding domain. The tyrosines in the chitin-binding domains, i.e. three in Pro-Resilin and five in CPR56F, probably do not participate at dityrosine formation as they are not accessible to cross-linking enzymes [17]. In total, potentially, a Pro-Resilin or Cpr56F tyrosine may be cross-linked to any of the 50 tyrosines of partner proteins. We assume that the strength of the DT signal in the microscope correlates with the number of DT per protein or matrix. Our data indicate a non-proportional relationship between Pro-Resilin and DT signal intensities in some Resilin regions. There are even Pro-Resilin-GFP patches, for instance in the femur, that seem to be almost DT-negative. This suggests that the activity of the peroxidase responsible for DT formation is controlled. This control is possibly important to define the elasticity of a Resilin matrix as elasticity correlates with the amount of DT [16, 17]. Biomechanics experiments should shed light on this issue.

DT formation activity seems not to be only under spatial but also under temporal control. In our experiments, we found that the DT signal intensified after eclosion when *pro-resilin* expression had ceased. This tentatively suggests that DT formation occurs within the extracellular space when Pro-Resilin is already incorporated in the cuticle. This is consistent with classical work published by Neville and Kristensen [41, 42]. Of course, this hypothesis needs to be tested at the protein level.

Variable region-specific cross-linking degree in Resilin matrices may inspire the analysis of cross-linking degree of tropoelastins in elastin matrices [43]. Indeed, covalent bonds between tropoelastins via lysinonorleucine, allysine aldol and desmosine are random [44] and, therefore, like in Resilin, may depend on the activity of cross-linking enzymes that in turn may be under tissue-specific control to adjust elastin elasticity to the needs.

### Pro-Resilin dysfunction is not lethal

As a flexible, resilient and adhesive element, Resilin is a multi-functional matrix in the cuticle of insects. In spite of this, we show that flies without Pro-Resilin, a key component of the Resilin matrix, are viable under laboratory conditions. In mice, deletions of the gene coding for the elastic protein Elastin lead to perinatal lethality [45]. In humans, heterozygous elastin deletions are, by contrast, not lethal, but cause severe supravalvular aortic stenosis (SVAS) or Williams-Beuren syndrome (WBS; omim.org).

Non-lethality in *D*. *melanogaster* is probably due to the presence of the potentially elastic protein Cpr56F that may compensate for Pro-Resilin deficiency. Cooperation or redundancy between Pro-Resilin and Cpr56F needs, of course, further, thorough analyses including the generation of double mutant clones in the adult body. Redundancy of elastic Resilin-forming proteins seems to be prevalent in insects. In the bed bug *Cimex lectularius*, 13 Pro-Resilin candidates have been described [46]. The *Anopheles gambiae* putative Pro-Resilin protein AgamCPR152 (317 residues) is shorter than the *D*. *melanogaster* Pro-Resilin protein (620 residues) [15], suggesting that more genes coding for elastic cuticle proteins may exist in the *A*. *gambiae* genome. We propose that the Resilin matrix may be composed of more than one elastic protein.

## Conclusions

In summary, our work supports the notion that the concept of “Resilin” involves more than one dityrosinylated protein. In line with a recent hypothesis [9], our results indicate that more than a Resilin protein is needed for construction of a Resilin-based elastic module in the insect cuticle. The cuticle of the fruit fly is subdivided into different regions of elasticity composed of different amounts of DT, Pro-Resilin and Cpr56F. It is possible that other Cpr proteins exist that cooperate with the here characterised elastic proteins to modulate yet variable degrees of elasticity. Taken together, according to our data, the Resilin concept has to be renewed: For the group of proteins that account for the elasticity and resilience of the cuticle, we coin the term Resilome; this does not consist of a single type of protein, Pro-Resilin, but, in *D*. *melanogaster*, of at least two proteins that are combined in different ratios to accommodate the respective specific function of the cuticle.

This finding will have an impact on the use of Resilin and the entire resilome in biomaterial sciences including micro-robotics [47–49]. As a composite material, the Resilin matrix is easily modifiable by changing the ratio between Pro-Resilin and Cpr56F. This possibility offers the opportunity to design and tailor Resilin-based materials for various technological and biomedical purposes by adjusting the elasticity to the specific needs.

## Methods

### Fly husbandry, genetics and transgenesis

Flies were kept in vials with standard cornmeal-based food at 18 or 25 °C. For embryo and larva collection, flies were kept in cages on apple-juice plates garnished with fresh baker’s yeast at 25 °C. Mutations were maintained over balancer chromosomes carrying insertions of GFP expressing marker genes (Dfd-YFP or Kr-GFP). This allowed us to identify homozygous non-GFP embryos or larvae as mutants under a standard fluorescence stereo-microscope (Leica).

For the feeding experiments, we chose to apply the CAFE assay [29]. In brief, ten flies of a given genotype that had been starved for 4–6 h were kept in a glass vial. Through a hole in the plastic lid of the vial, a glass capillary containing a defined column of apple juice was inserted to allow drinking. After 3 h, the amount of ingested apple juice was determined in percent. The experiment was repeated at least six times.

The transposon plasmids with either the *pro-resilin* (41.9 kb) or *cpr56F* (33.2 kb) genomic DNA and the *gfp*-coding region fused to the 3′ end of *pro-resilin* or *cpr56F* were obtained from Source Bioscience (https://www.sourcebioscience.com) (Additional file 1: Fig. S1). Transgenic flies harbouring these transposons inserted on the left arm of chromosome 2, PBac (yellow[+]-attP-9A)VK00014, were generated by BestGene (www.thebestgene.com).

To recombine the *pro-resilin* coding region with the coding region of Venus downstream of the UAS promoter, the *pro-resilin* coding region was cloned into the pTWV transposon plasmid obtained from the Drosophila Genomic Resource Center (DGRC). Transgenic flies (*w*^*1118*^) with this UAS-*pro-resilin-venus* construct were generated by BestGene. For *pro-resilin-venus* expression, we used the ubiquitous *da*-Gal4 driver.

For RNA interference (RNAi), the hairpin RNA constructs 106773 (against *pro-resilin*), 13296 and 110265 (both against *cpr56F*) from the Vienna Drosophila Resource Center (VDRC) were expressed using the ubiquitous *da*-Gal4 driver.

### Crispr/Cas9

To randomly mutate the *pro-resilin* gene in *D*. *melanogaster*, we followed the protocol at the flyCRISPR homepage (http://flycrispr.molbio.wisc.edu) developed at the Wisconsin University. For *pro-resilin*, we designed two gDNA g4 (GACGCTGCTCATGGCAATGGTGG) and g5 (GGGCATTCAGAGATGCGCGACGG) that are complementary to sequences in exon 1 and exon 2, respectively. For *cpr56F*, one gDNA (GAGGCAGCCAGGCACACGAGC) was designed. According to the protocol at the flyCRISPR homepage, complementary oligos with appropriate overhangs were annealed and cloned into the pCFD3-dU6/3 vector at the BbsI site. After amplification in bacteria, these vectors were injected separately into Cas9 expressing embryos (Bloomington Stock Number 54590: y [1] M(w[+mC] = Act5C-Cas9.P)ZH-2A w[*]). Injected embryos developed to flies that were crossed en masse to partners harbouring the CyO balancer. Single males of the progeny of this cross were backcrossed to CyO [Dfd-YFP] flies to establish stocks. Homozygous non-CyO flies were examined for phenotypes. To identify any mutation, exon 1 and 2 were sequenced in the genomic DNA of non-YFP larvae of these stocks.

### Histology, microscopy and imaging

A Leica DMI8 equipped with a Hamamatsu Orca R2 camera was used for whole-mount signal detection in Figs. 1, 3, 5 and 8 and Additional files 3, 5, 7 and 16. The settings were: GFP: excitation [Ex] with a 450-490 nm light source, detection through a 500–550-nm filter; dark Red (Y5): Ex: 590-650 nm, dichroic filter (DC): 660 nm, emission [Em]: 662-738 nm; DAPI: Ex: 325-375 nm, DC: 400 nm, Em: 435-485 nm and rhodamine: Ex: 541-551 nm, DC: 560 nm, Em:565-605 nm with an exposure time of 400 ms for fluorescence imaging. Bright-field (no filter) images were obtained with an exposure time of 25 ms. The objectives were as follows: HC FL PLAN 5x/0.12 DRY, HC PL FLUOTAR 10x/0.30 DRY, HC PL FLUOTAR L 20x/0.40 DRY and HC PL FLUOTAR L 40x/0.60 DRY. For the observation of signal localisation and intensity comparison by fluorescence microscopy, 150 individuals were used.

A Leica M205FA fluorescence binocular equipped with a DFC3000G-0055483516 camera was used. Images are shown in Additional file 14. An LED-source was used for bright-field and GFP (long pass filter with Ex: 460–500 nm, Em: > 510 nm) imaging with an exposure time of 400 ms.

We also used a fluorescence microscope Axio Observer Z1 with an Axiocam Mono camera. Images are shown in Additional file 14: Figure S14. The exposure time was set to 5.2 s. The filters were as follows: AF405 (DT specific filter): Ex: 357/44 nm Brightline HC, splitter HC BS 389 nm, Em: 420/40 nm ET Bandpass; DAPI: Ex: 359/48 nm, splitter: 395 nm, Em: 445/50 nm and GFP: Ex: 470/40 nm, splitter: 495 nm, Em: 525/50 nm. The objectives were EC Plan-Neofluar 5x/0.67 M27 DRY, EC Plan-Neofluar 10x/0.3 M27 DRY and EC Plan-Neofluar 20x/0.5 M27 DRY. As a light source, a halogen lamp was used. For this experiment, 15 individuals were observed.

A Zeiss LSM880 was used for GFP and DT detection. Images are shown in Figs. 2 and 4 and Additional files 4 and 11. Two different modes of microscopy were operated on this microscope. A normal confocal mode with the settings: GFP images: no intensity of the laser given, Ex: 488 nm and Em: 548 nm, pixel integration: 2.125 μs, pinhole 1.63 AU/33 μm; 405 autofluorescence images: Ex: 405 nm and Em: 464 nm, pinhole 1.93 AU/33 μm; DT images: Ex: 355 nm and Em: 415 nm, pinhole 2.16 AU/33 μm, pixel integration 1.03 μs. And a fast airyscan mode with the settings: Ex (with a laser intensity of 1.5%) for DT: 355 nm and for the 405 autofluorescence: 405 nm and Em for both: 420 nm, pinhole 11.19 AU for both. The objectives were Plan-Apochromat 10x/0.45 M27 DRY and 20x/0.8 M27 DRY.

For quantitative data generation, signal intensities were measured and compared using intensity data determined with the Fiji software. To obtain a DT-specific signal for Resilin detection, the interfering background signal excited by the 405 nm light source had to be eliminated. This background signal emanates partly from DT, partly from the outermost cuticle layer called envelope [50, 51]. For this purpose, i.e. elimination of the background, we calculated the mean difference between the 355 nm and the 405 nm signal intensities in the wing articulation of *hdw* flies, which was around 3,000,000. For a higher stringency, however, we chose to continue our analyses with the threshold of 4,000,000 that was defined henceforth as the threshold pixel intensity value. Signals above this threshold were considered as valid. Lowering the threshold to 1,000,000 yielded only about 10% more valid signals than the operational threshold. The defined threshold for the signal in the wing articulation was applied to the trochanter. To observe differences in DT signal intensities in wild-type and *hdw* flies and to examine the variation between 2 days old and freshly eclosed adults, 35 individuals for the wing articulation and 62 individuals for the legs were dissected (preparation in AquaPolymount). Optical sections were generated with the normal confocal mode yielding 28 z-stack images that were subsequently processed with the Fiji software. Two different modes of validation were implemented in order to possibly detect reasonable DT signal differences between wild-type and *hdw* flies. In the first approach, we tested how often the signal intensity threshold of 4,000,000 that was determined initially was reached in wild-type control and *pro-resilin* deficient flies (*hdw*). The positive signal incidence in the wild-type control was set to 100%; the positive signal incidence in *hdw* flies is given as a fraction thereof. In the second approach, the intensity values of wild-type control and *pro-resilin* deficient fly were directly compared statistically; these data are shown for the area and the intensity excited at 355 nm in Fig. 6a and b. The mean values with the standard deviations are shown in the figure. The significance was calculated by a two-sided Student’s *t* test.

A Nikon AZ100 zoom microscope operated with the NIS Elements D software was used for Nomarski (DIC) imaging of wing articulations shown in Additional file 6: Figure S6. Ten flies of each genotype were anesthetised with carbon dioxide, submerged in Halocarbon oil 700 (Sigma-Aldrich) and covered with a coverslip for microscopy (gain 1, exposure time 14 ms).

The phenotype of live adult flies (*n* = 20) in a well plate was recorded by a Leica EZ4HD with an in-built camera (Additional file 16: Movie S1). Flight of flies (*pro-resilin*^*RNAi*^ *n* = 35; wild-type *n* = 6) fixed with Clean Glass adhesive from Duro on a needle with a diameter of 127 μm was filmed with an u3 cmos 14000KPA microscope eyepiece camera (Additional files 17 and 18: Movies S2 and 3). The behaviour of flies in a well plate containing a filter paper and dry yeast was filmed using a DNT DigiMicro 2.0 Scale Digital Mikroskop (Additional files 19, 20, 21, 22, 23, 24: Movies S4, S5, S6, S7, S8, S9). Over 100 flies of each genotype were studied.

Eosin Y staining was conducted according to the recently published protocol [28]. In brief, flies (*n* = 10, two biological replicates) were incubated with Eosin Y (0.5%, Sigma-Aldrich) at 25 °C for 20 min, washed with tap water and deposited on a slide for microscopy and imaging (Leica EZ4HD with an in-built camera).

Figures were prepared and assembled using the Microsoft PowerPoint and Excel, Inkscape (www.inkscape.org), Leica LAS X, ZenBlue, Adobe Illustrator and Photoshop CS6 and GIMP-2.10 software. For the images shown in Figs. 2, 3 and Additional file 11: Figure S11, artificial digital pixel intensity values of 0 for black and 12,000 for white were used.

### Molecular biology

Cloning of recombinant DNA, PCR and sequencing were performed following standard laboratory methods. For transcript quantification by quantitative real-time PCR (qPCR), RNA was first isolated from flies using the RNEasy kit (Qiagen) and used for cDNA synthesis (High-Capacity cDNA Reverse Transcription Kit nr. 4368814, Thermo Fisher Scientific). qPCR was performed according to the Sybr-Green method (SYBR® Green Master Mix, nr. A25741 Thermo Fisher Scientific) on a QuantStudio 5 cycler (Thermo Fisher Scientific). Data were analysed with the in-built software (Quantstudio5). Expression of *pro-resilin* and *cpr56F* were normalised against the expression of *rpl32* and *rps20*. The quantification experiments were performed according to the MIQE guidelines [52]. Experiments were performed twice (technical replicates) with 5 individuals of each line and age; each experiment was repeated three times (biological replicates, i.e. independent fly collections). In Fig. 7 and Additional file 13: Figure S13, the 95% confidence intervals are shown in a logarithmical scale. In Additional file 2: Figure S2, the mean values and the standard deviation were calculated using Microsoft Excel.

## Supplementary Information

**Additional file 1: Figure S1.** Upper scheme: The transposon harbouring the genomic region of the *pro-resilin* gene (orange) fused with the ORF of sGFP (green) at its 3′ end before the stop codon encompasses 27,324 bps of upstream and 10,204 bps of downstream sequences. Lower scheme: The transposon harbouring the genomic region of the *cpr56F* gene (orange) fused with the ORF of sGFP (green) at its 3′ end before the stop codon encompasses 13,674 bps of upstream and 15,487 bps of downstream sequences.

**Additional file 2: Figure S2.** Real-time quantitative PCR analysis indicates that *pro-resilin* (*res*) is not expressed before the pupal stage. The expression of the house-keeping gene *Rps20* was used to normalize the expression data. In addition, we used a primer pair that does not amplify any DNA in the fly transcriptome as a negative control (neg). The expression value for this amplification was set to 1. The amplification levels of *pro-resilin* (fold change with respect to the negative control) were identical to the amplification levels of this primers in embryonic and larval samples. In pupae, *pro-resilin* expression is clearly induced. Bars indicates standard deviation.

**Additional file 3: Figure S3.** A–C: Leg and adjacent thoracic wall of a *D*. *melanogaster*. Blue labels “l + number” (l leg) specify the various Resilin patches (red signal) found; see Additional file 25 for exact morphological location and discussion of patches. (A) Entire leg, (B) distal part of tibia and proximal part of tarsus enlarged and (C) subsequent tarsomeres enlarged. Further labelling: cl … claws, co... coxa, fr... femur, tb... tibia, th... trochanter, tr... tarsus with tarsomeres 1–5, txs... thorax, exact location of signal unclear. (D) Wing base and adjacent parts of the mesothorax (left side) of a *D*. *melanogaster*. Blue labels “w + number” (w wing, wa wing articulation) specify the various Resilin patches (red signals) found. Upcal upper calyptere; Sc, R, MA, CuA1, A1 are wing veins, and P1–5 are wing articulation sclerites (pteralia; ? indicates an ambiguous identification) using the standard terminologies.

**Additional file 4: Figure S4.** In the tracheal endings (sp) and the bristle sockets (br, 2 are visible), very weak DT signals (A and C) overlap with the Pro-Resilin-GFP signal (A and B). The asterisk (*) marks auto-fluorescence of internal tissues after dissection. Images were generated on a Zeiss LSM880 confocal microscope. The excitation (ex) and emission (em) wavelengths are indicated in the images that were obtained by the normal confocal mode. Details of the settings are described in the Methods section.

**Additional file 5: Figure S5.** Pro-Resilin-GFP in spermatheca. (A) In the spermathecal ducts of freshly eclosed females, we observe a weak Pro-Resilin-GFP signal (black arrows). (B) This signal becomes stronger in one-day-old flies and (C) well visible in seven-day-old females. (D) The spermathecal ducts of freshly eclosed, (E) one-day-old or (F) seven-day-old females with reduced *pro-resilin* expression (*pro-resilin*^*RNAi*^) appear to be normal. The GFP signal (red) was merged with the bright-field image. A Leica DMi8 microscope was used for imaging. Details of the settings are described in the Methods section.

**Additional file 6: Figure S6.** The morphology of the wing articulation region may depend on Resilin. The wild-type wing (w) articulation region in the thorax is framed by the anepisternum (x) and the anepimeron (*). It is composed of three sclerites (1–3) and the pleural wing process. The sclerites 1 and 2 are separated by the flexible vertical cleft (arrow). In the wild-type wing articulation regions, we spotted a triangular structure (triangle) that is missing in the *pro-resilin*^*CC-ATG*^ fly. The wing articulation elements were named according to a reproduction of the thorax at flybase (http://flybase.org/reports/FBim0000793). Images were recorded on a Nikon AZ100 zoom microscope applying Nomarski microscopy.

**Additional file 7: Figure S7.** Pro-Resilin-GFP expression (green signal) normalizes the *hdw* phenotype of *pro-resilin*^*RNAi*^ flies. The fly has been illuminated additionally with green light (rhodamine filter, red signal) and UV light (DAPI, blue signal). All colours have been merged with a bright-field (BF) image with the aim to show a plastic image of the fly taken with a Leica DMI8 microscope. Details of the settings are described in the Methods section. The image was digitally manipulated without changing the ratios of the intensities of the different signals.

**Additional file 8: Figure S8.** Schematic representation of the *pro-resilin* gene and its products. (A) The full-length Pro-Resilin protein has 620 residues. It has three functional domains. Following the signal peptide (SP, white box) in the N-terminus, there are a number of tandem repeats (type A repeats, dark grey boxes), followed by an R&R-2 chitin-binding domain, which precedes an array of tandem repeats (type B repeats, light grey boxes). A shorter isoform lacks the R&R-2 domain and has 575 residues (not shown). Deletion of 3 bases in the second exon coding for the R&R-2 domain results in the transversion of the M^365^ to an I and the deletion of R^366^ as shown above the full-length Pro-Resilin protein (Crispr/Cas9^2^). Deletion of the cytosine 35 and adenosine 36 of the ORF causes a frameshift of the ORF resulting in a premature stop codon after 22 residues (Pro-Resilin^CC-ATG^). Information on the mutations at the DNA level is presented in Additional file 10: Figure S10. (B) The *pro-resilin* gene is composed of three exons separated by two introns. Exon 1 and 3 can be spliced together omitting exon 2 that encodes the R&R-2 domain. The recognition sites of hairpin RNAs against *pro-resilin* expression are indicated below the scheme. (C) The GFP coding region is inserted in frame into the 5′ end of the *pro-resilin* gene just before the stop codon, thereby disrupting the recognition site of hairpin RNAs.

**Additional file 9: Figure S9.** Schematic representation of the *cpr56F* gene and its product. (A) The *cpr56F* gene has three exons and two introns. (B) The GFP coding region is inserted in frame into the 5′ end of the *cpr56F* gene just before the stop codon, thereby disrupting the recognition site of one hairpin RNA (KK), without affecting the other one (GD). (C) Cpr56F (217 residues), is composed of a signal peptide (SP, white) and two repeats (grey) separated by an R&R-2 domain (black). We generated two Crispr/Cas9 induced *cpr56F* mutant alleles. One, *cpr56*^*12*^ gives rise to a protein with a deletion of three amino acids in its signal peptide. The other allele, *cpr56*^*2*^, is characterised by the deletion of cytosine 30 in the ORF resulting in a frameshift. The respective protein has 88 amino acids including 78 residues absent in the normal Cpr56 protein (light grey region). Information on the mutations at the DNA level is presented in Additional file 10: Figure S10.

**Additional file 10: Figure S10.** (A) The *pro-resilin* locus, genomic organisation and mutations. The two bases CA (green box) in the first exon (magenta letters) are deleted in the *pro-resilin*^*CC-ATG*^ allele. This causes a frameshift and a premature stop codon resulting in a truncated protein as shown in Additional file 8: Figure S8. The three bases GCG (blue box) in the second exon (magenta letters) are deleted in the *pro-resilin*^*CC-RR*^ allele. By this deletion that affects two consecutive codons, the amino acid M^365^ is changed to an I and the amino acid R^366^ is deleted as shown in Additional file 8: Figure S8. (B) The *cpr56F* locus, genomic organisation and mutations. The nine bases CGTGTCCCT (underlined green box) in the second exon (magenta letters) are deleted in the *cpr56F*^*12*^ allele. This causes a deletion of the three amino acids LVC in the signal peptide of the resulting protein as shown in Additional file 9: Figure S9. The cytosine^2458^ (framed box) in the second exon (magenta letters) is deleted in the *cpr56F*^*2*^ allele. This deletion causes a frameshift resulting in an aberrant protein sequence of 78 amino acids after L^10^ and a premature stop codon as shown in Additional file 9: Figure S9.

**Additional file 11: Figure S11.** (A-A″) Pro-Resilin-GFP is predominantly detected in or close to the leg joints (see also Fig. 1d). (A″) An auto-fluorescent signal derived from DT overlaps with the Pro-Resilin-GFP signal. (B) In wild-type samples, the signals in the legs are similar to those in Pro-Resilin-GFP legs. As shown in Fig. 3, a DT signal in the femur close to the tibia-femur joint corresponding to a strong Pro-Resilin-GFP (not visible in A, see Fig. 3a) is missing. In *pro-resilin*^*RNAi*^ (C) and *pro-resilin*^*cc-ATG*^ (D) samples, the DT signal is not strongly reduced. Differences in intensity are, however, apparent upon software-based anaylses (Fig. 6). Images were generated with a Zeiss LSM880 confocal microscope. The excitation (ex) and emission (em) wavelengths are indicated in the images. Those shown in A-A″ were obtained by the normal confocal mode, while those shown in B-D were produced by the fast airyscan mode. Details of the respective settings are described in the Methods section. Labelling: co … coxa, fr … femur, tb … tibia, th … trochanter. The asterisk (*) marks auto-fluorescence of internal tissues after dissection.

**Additional file 12: Figure S12.** The tracheal systems of wild-type and *resilin*^*cc-ATG*^ flies are unstained after incubation with Eosin Y (red) that, by contrast, penetrates the tracheal system of *parched*^*22*^ flies (triangles), which have been described to have open spiracles [39]. Images were generated with a Leica EZ4HD with an in-built camera.

**Additional file 13: Figure S13.** Reduction of Cpr56F does not cause any visible phenotype. (A) When resting, the wild-type fly holds its wings at the back. (B, B′) RNAi against *cpr56F* does not have any effect on wing posture. (C) Some flies with reduced *cpr56F* expression have crippled wings. (D) *cpr56F* is highly expressed in pupae. Its expression drops in eclosed flies. *cpr56F* expression is not strongly reduced by RNAi. According to the overlapping confidence intervals (CI), the differences in expression levels are not highly significant. Images were generated with a Leica EZ4HD with an in-built camera.

**Additional file 14: Figure S14.** Cpr56F-GFP is detected in the head region of the embryo and larva. (A) The head skeleton and the tip of the head of the ready-to-hatch embryo contain Cpr56F-GFP. (B) This signal persists during larval stages. Images were generated with a Leica M205FA fluorescence binocular. Details of the respective settings are described in the Methods section. Labelling: lsg... larval salivary gland, ci...(larval) cibarium, lb... (larval) labellum.

**Additional file 15: Figure S15.** The proximal edge of the marginal cell (mc) in the wing of *Drosophila hydei* contains dityrosine (A). In the green channel, this signal is missing (B). Cpr56F-GFP is expressed at this position (C), whereas Pro-Resilin-GFP is not (see Fig. 1). The region of dityrosine and Cpr56F-GFP signal in the wing was named according to a reproduction of the wing at flybase (http://flybase.org/reports/FBim0000833). Here, we used an Axio Observer Z1 microscope for imaging. The excitation (ex) and emission (em) wavelengths are indicated in the images. Details of the respective settings are described in the Methods section.

**Additional file 25: Text.** Extended description and discussion of Resilin patches in the fly leg.

## Data Availability

All data generated or analysed during this study are included in this published article and its supplementary information files.
