# Correction to: Resilin matrix distribution, variability and function in *Drosophila*

**DOI:** 10.1186/s12915-021-01090-5

**Published:** 2021-07-30

**Authors:** Steven Lerch, Renata Zuber, Nicole Gehring, Yiwen Wang, Barbara Eckel, Klaus-Dieter Klass, Fritz-Olaf Lehmann, Bernard Moussian

**Affiliations:** 1 Applied Zoology, Technical University of Dresden, Dresden, Germany; 2 Animal Genetics, Interfaculty Institute of Cell Biology, University of Tübingen, Tübingen, Germany; 3 Senckenberg Natural History Collections, Dresden, Germany; 4 Animal Physiology, University of Rostock, Rostock, Germany; 5 CNRS, Inserm Institute of Biology Valrose, Université Côte d’Azur, Nice, France

**Correction to: BMC Biol 18, 195 (2020)**

**https://doi.org/10.1186/s12915-020-00902-4**

Following publication of the original article [1], an error was identified in reference 23. It should have been presented as: Rice MJ. Function of resilin in tsetse fly feeding mechanism. Nature. 1970;228(5278):1337–8.

The original article [1] has been corrected.
