# Supplementary figures and images for: Resilin matrix distribution, variability and function in Drosophila
Source: BMC Biol. 2020 Dec 14;18:195. doi: 10.1186/s12915-020-00902-4 (PMC7737337; doi:10.1186/s12915-020-00902-4)

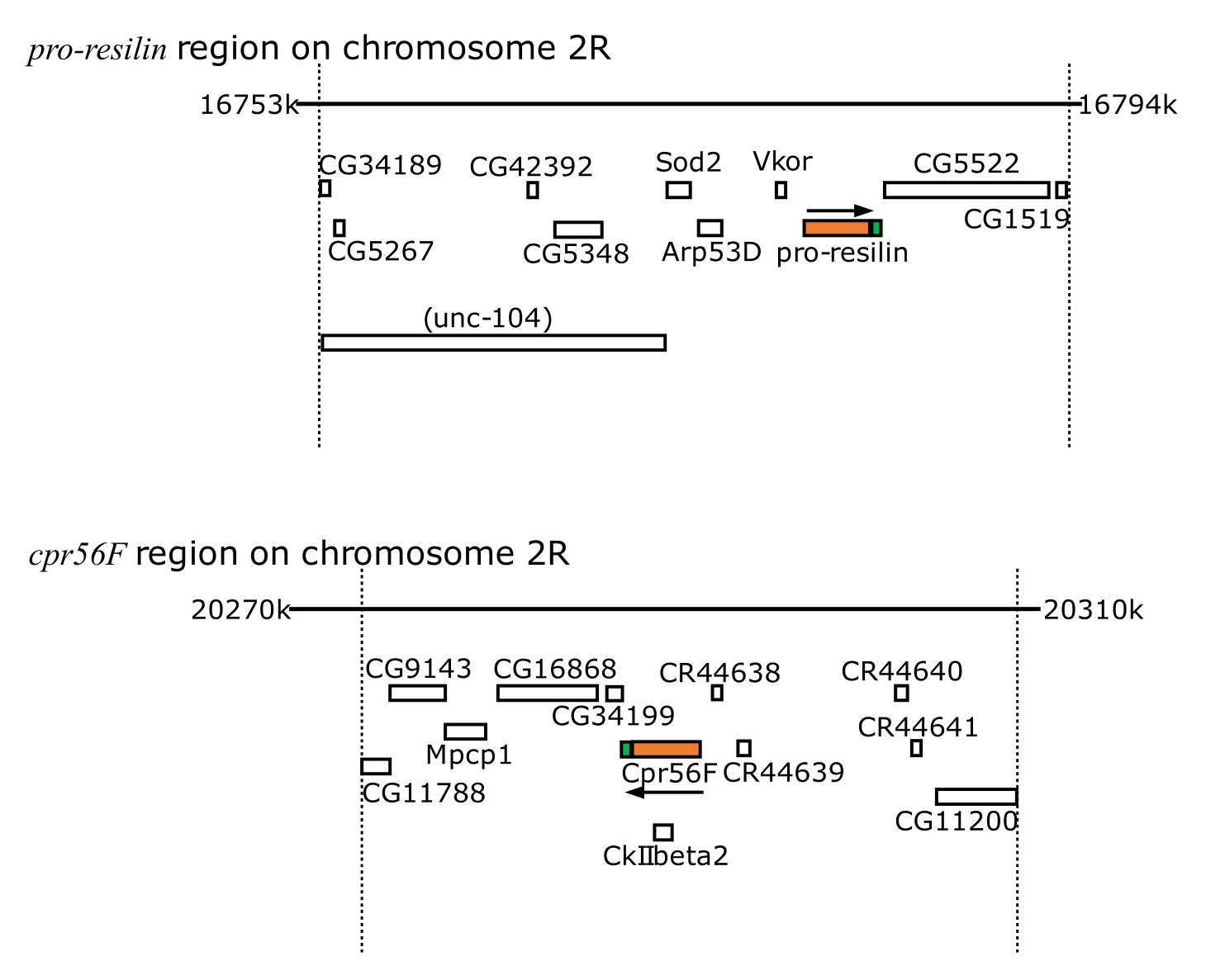

Supplement: Supplementary file 10 — Additional file 1: Figure S1. Upper scheme: The transposon harbouring the genomic region of the pro-resilin gene (orange) fused with the ORF of sGFP (green) at its 3′ end before the stop codon encompasses 27,324 bps of upstream and 10,204 bps of downstream sequences. Lower scheme: The transposon harbouring the genomic region of the cpr56F gene (orange) fused with the ORF of sGFP (green) at its 3′ end before the stop codon encompasses 13,674 bps of upstream and 15,487 bps of downstream sequences. [file 12915_2020_902_MOESM1_ESM.jpg]

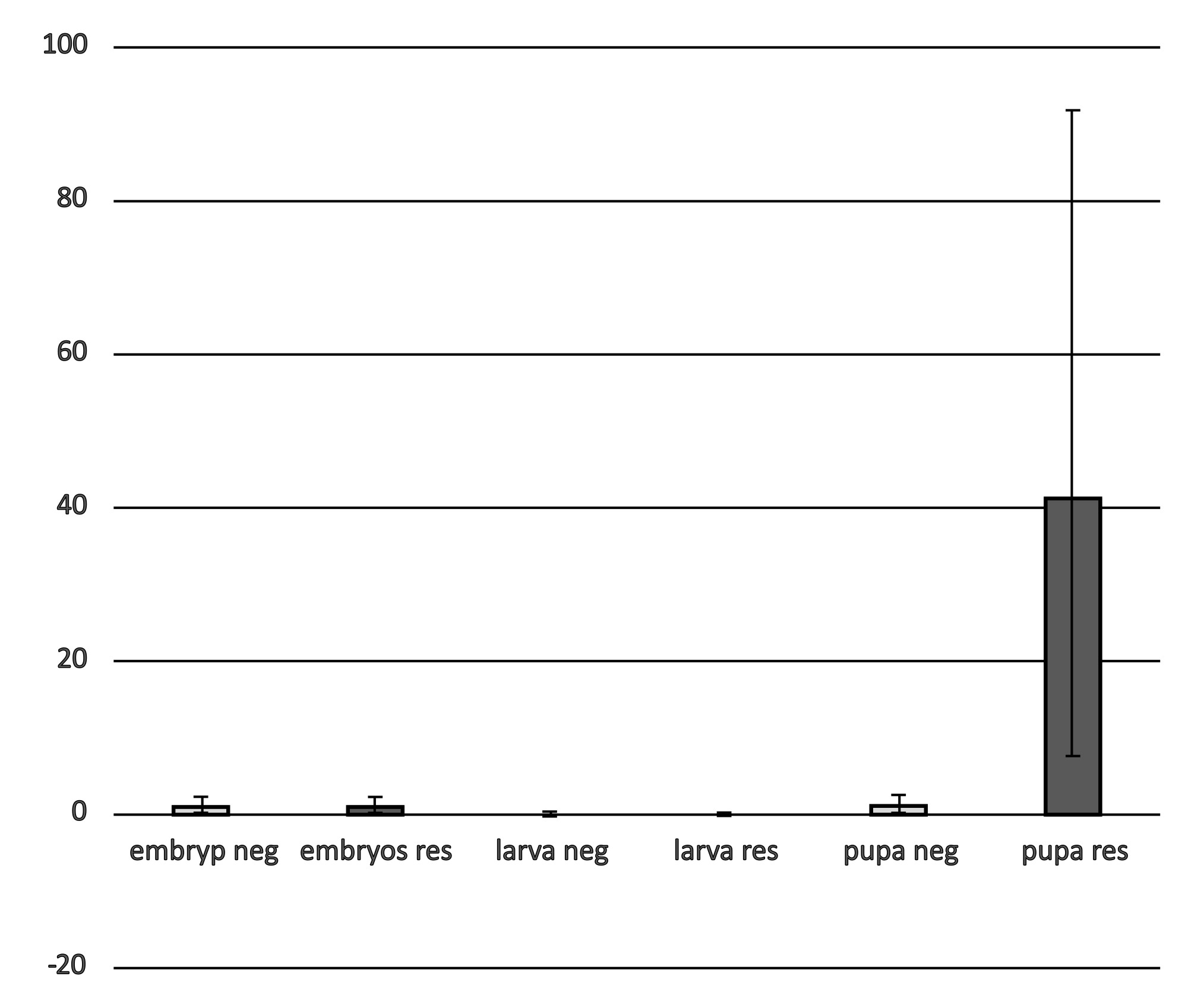

Supplement: Supplementary file 11 — Additional file 2: Figure S2. Real-time quantitative PCR analysis indicates that pro-resilin (res) is not expressed before the pupal stage. The expression of the house-keeping gene Rps20 was used to normalize the expression data. In addition, we used a primer pair that does not amplify any DNA in the fly transcriptome as a negative control (neg). The expression value for this amplification was set to 1. The amplification levels of pro-resilin (fold change with respect to the negative control) were identical to the amplification levels of this primers in embryonic and larval samples. In pupae, pro-resilin expression is clearly induced. Bars indicates standard deviation. [file 12915_2020_902_MOESM2_ESM.jpg]

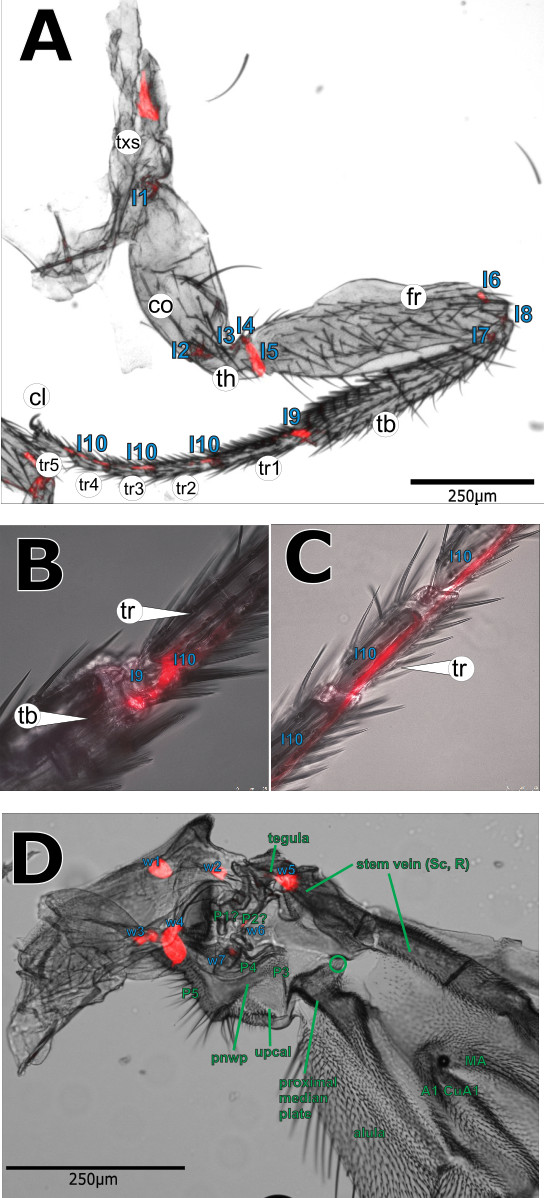

Supplement: Supplementary file 12 — Additional file 3: Figure S3. A–C: Leg and adjacent thoracic wall of a D. melanogaster. Blue labels “l + number” (l leg) specify the various Resilin patches (red signal) found; see Additional file 25 for exact morphological location and discussion of patches. (A) Entire leg, (B) distal part of tibia and proximal part of tarsus enlarged and (C) subsequent tarsomeres enlarged. Further labelling: cl … claws, co... coxa, fr... femur, tb... tibia, th... trochanter, tr... tarsus with tarsomeres 1–5, txs... thorax, exact location of signal unclear. (D) Wing base and adjacent parts of the mesothorax (left side) of a D. melanogaster. Blue labels “w + number” (w wing, wa wing articulation) specify the various Resilin patches (red signals) found. Upcal upper calyptere; Sc, R, MA, CuA1, A1 are wing veins, and P1–5 are wing articulation sclerites (pteralia; ? indicates an ambiguous identification) using the standard terminologies. [file 12915_2020_902_MOESM3_ESM.jpg]

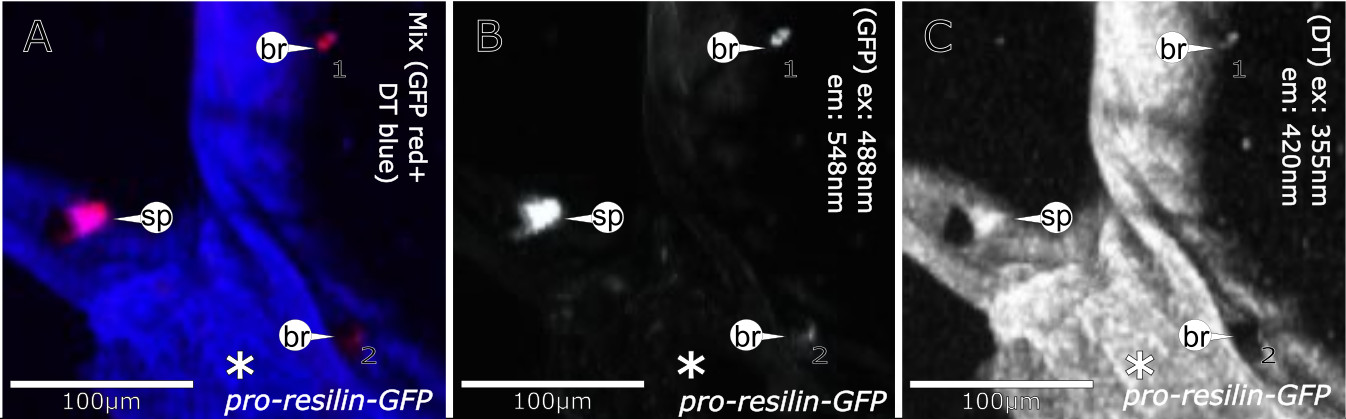

Supplement: Supplementary file 13 — Additional file 4: Figure S4. In the tracheal endings (sp) and the bristle sockets (br, 2 are visible), very weak DT signals (A and C) overlap with the Pro-Resilin-GFP signal (A and B). The asterisk (*) marks auto-fluorescence of internal tissues after dissection. Images were generated on a Zeiss LSM880 confocal microscope. The excitation (ex) and emission (em) wavelengths are indicated in the images that were obtained by the normal confocal mode. Details of the settings are described in the Methods section. [file 12915_2020_902_MOESM4_ESM.jpg]

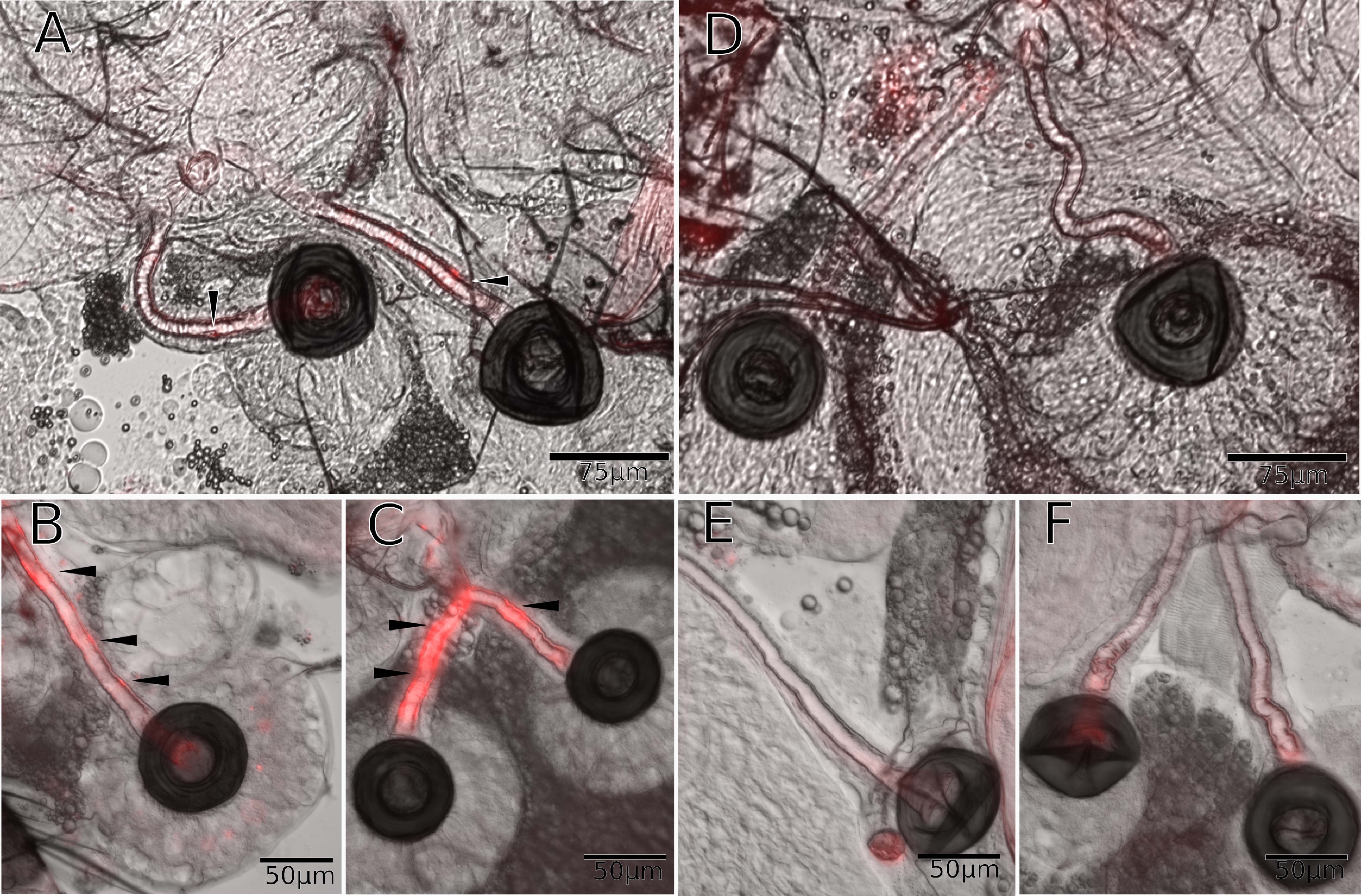

Supplement: Supplementary file 14 — Additional file 5: Figure S5. Pro-Resilin-GFP in spermatheca. (A) In the spermathecal ducts of freshly eclosed females, we observe a weak Pro-Resilin-GFP signal (black arrows). (B) This signal becomes stronger in one-day-old flies and (C) well visible in seven-day-old females. (D) The spermathecal ducts of freshly eclosed, (E) one-day-old or (F) seven-day-old females with reduced pro-resilin expression (pro-resilinRNAi) appear to be normal. The GFP signal (red) was merged with the bright-field image. A Leica DMi8 microscope was used for imaging. Details of the settings are described in the Methods section. [file 12915_2020_902_MOESM5_ESM.jpg]

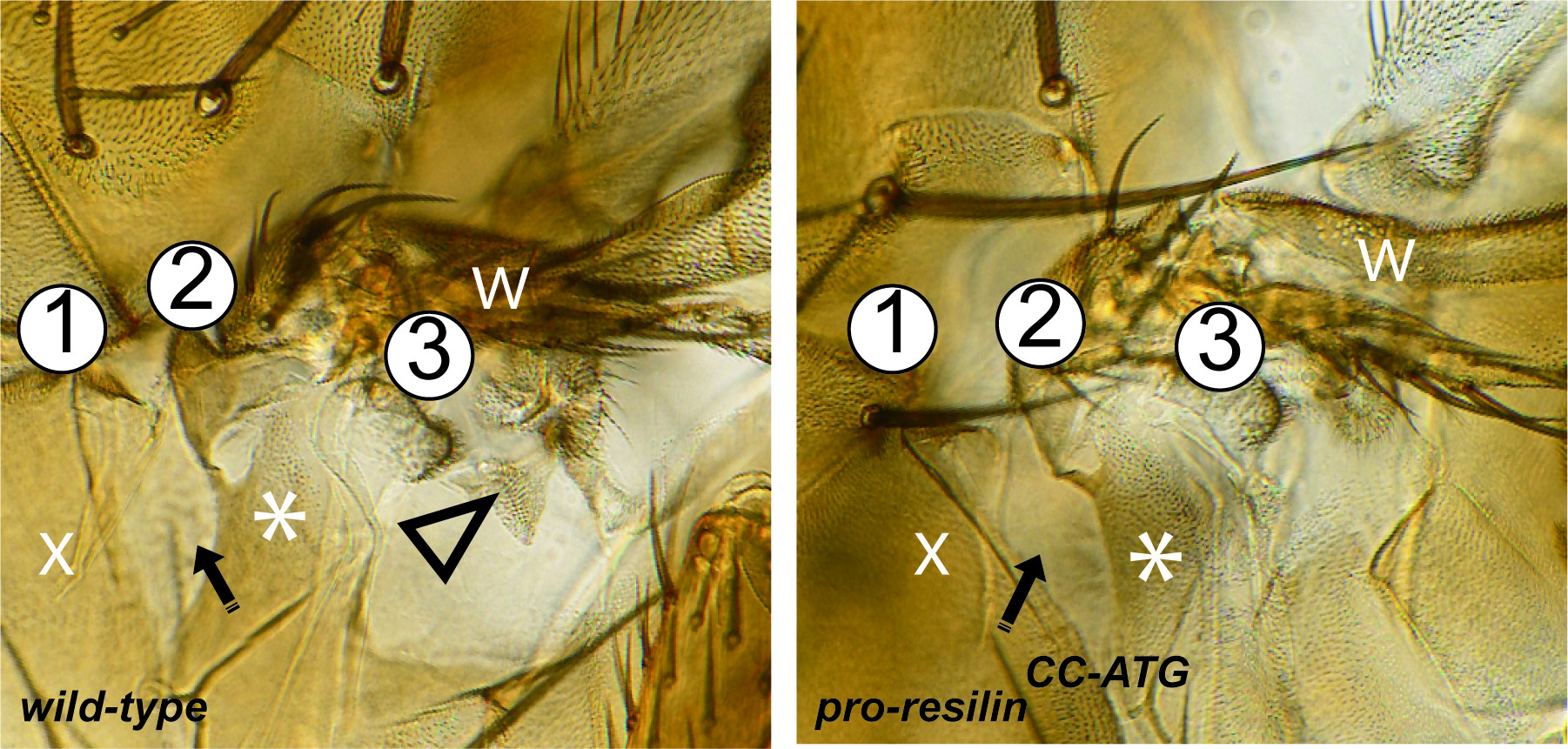

Supplement: Supplementary file 15 — Additional file 6: Figure S6. The morphology of the wing articulation region may depend on Resilin. The wild-type wing (w) articulation region in the thorax is framed by the anepisternum (x) and the anepimeron (*). It is composed of three sclerites (1–3) and the pleural wing process. The sclerites 1 and 2 are separated by the flexible vertical cleft (arrow). In the wild-type wing articulation regions, we spotted a triangular structure (triangle) that is missing in the pro-resilinCC-ATG fly. The wing articulation elements were named according to a reproduction of the thorax at flybase (http://flybase.org/reports/FBim0000793). Images were recorded on a Nikon AZ100 zoom microscope applying Nomarski microscopy. [file 12915_2020_902_MOESM6_ESM.jpg]

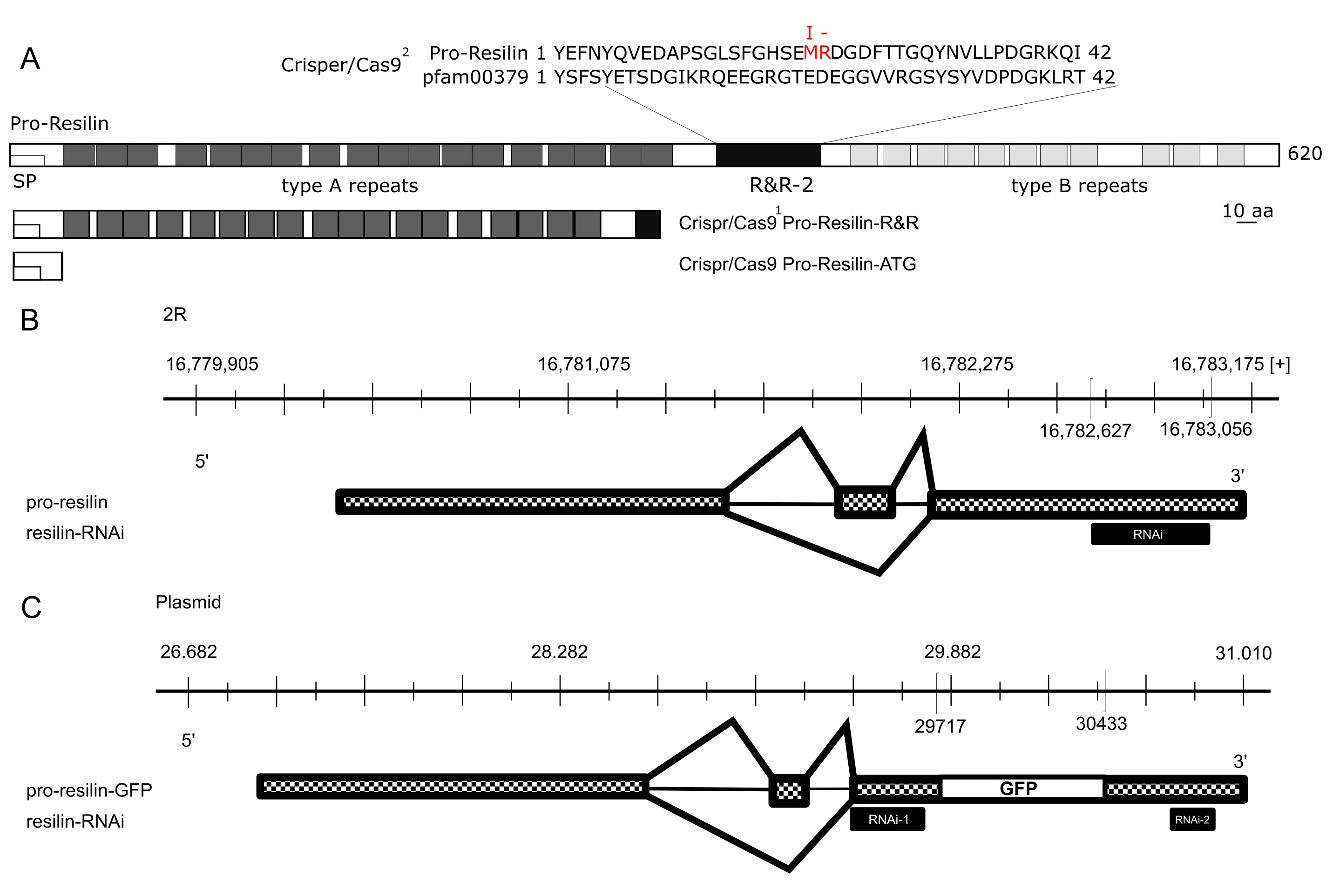

Supplement: Supplementary file 17 — Additional file 8: Figure S8. Schematic representation of the pro-resilin gene and its products. (A) The full-length Pro-Resilin protein has 620 residues. It has three functional domains. Following the signal peptide (SP, white box) in the N-terminus, there are a number of tandem repeats (type A repeats, dark grey boxes), followed by an R&R-2 chitin-binding domain, which precedes an array of tandem repeats (type B repeats, light grey boxes). A shorter isoform lacks the R&R-2 domain and has 575 residues (not shown). Deletion of 3 bases in the second exon coding for the R&R-2 domain results in the transversion of the M365 to an I and the deletion of R366 as shown above the full-length Pro-Resilin protein (Crispr/Cas92). Deletion of the cytosine 35 and adenosine 36 of the ORF causes a frameshift of the ORF resulting in a premature stop codon after 22 residues (Pro-ResilinCC-ATG). Information on the mutations at the DNA level is presented in Additional file 10: Figure S10. (B) The pro-resilin gene is composed of three exons separated by two introns. Exon 1 and 3 can be spliced together omitting exon 2 that encodes the R&R-2 domain. The recognition sites of hairpin RNAs against pro-resilin expression are indicated below the scheme. (C) The GFP coding region is inserted in frame into the 5′ end of the pro-resilin gene just before the stop codon, thereby disrupting the recognition site of hairpin RNAs. [file 12915_2020_902_MOESM8_ESM.jpg]

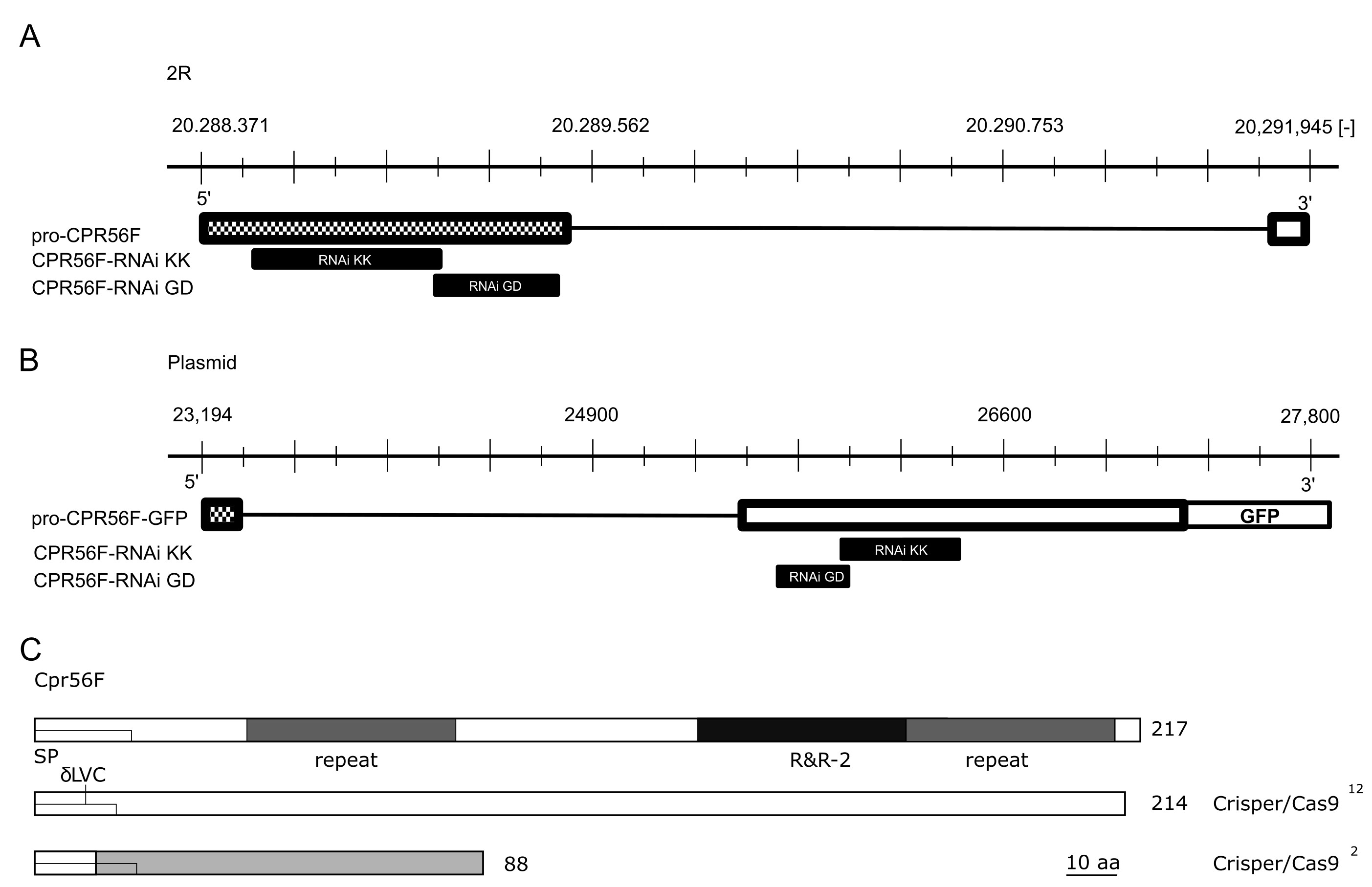

Supplement: Supplementary file 18 — Additional file 9: Figure S9. Schematic representation of the cpr56F gene and its product. (A) The cpr56F gene has three exons and two introns. (B) The GFP coding region is inserted in frame into the 5′ end of the cpr56F gene just before the stop codon, thereby disrupting the recognition site of one hairpin RNA (KK), without affecting the other one (GD). (C) Cpr56F (217 residues), is composed of a signal peptide (SP, white) and two repeats (grey) separated by an R&R-2 domain (black). We generated two Crispr/Cas9 induced cpr56F mutant alleles. One, cpr5612 gives rise to a protein with a deletion of three amino acids in its signal peptide. The other allele, cpr562, is characterised by the deletion of cytosine 30 in the ORF resulting in a frameshift. The respective protein has 88 amino acids including 78 residues absent in the normal Cpr56 protein (light grey region). Information on the mutations at the DNA level is presented in Additional file 10: Figure S10. [file 12915_2020_902_MOESM9_ESM.jpg]

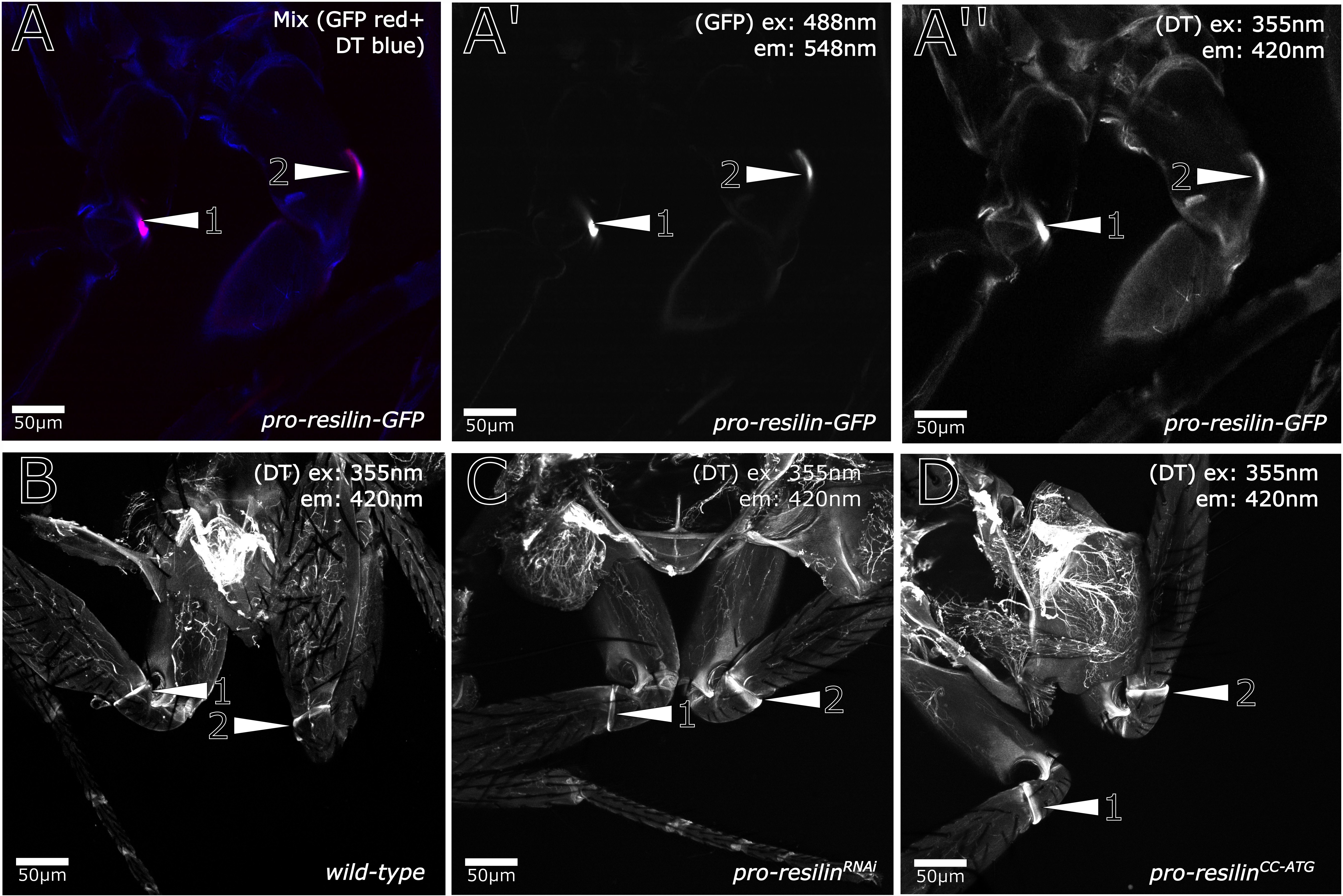

Supplement: Supplementary file 20 — Additional file 11: Figure S11. (A-A″) Pro-Resilin-GFP is predominantly detected in or close to the leg joints (see also Fig. 1d). (A″) An auto-fluorescent signal derived from DT overlaps with the Pro-Resilin-GFP signal. (B) In wild-type samples, the signals in the legs are similar to those in Pro-Resilin-GFP legs. As shown in Fig. 3, a DT signal in the femur close to the tibia-femur joint corresponding to a strong Pro-Resilin-GFP (not visible in A, see Fig. 3a) is missing. In pro-resilinRNAi (C) and pro-resilincc-ATG (D) samples, the DT signal is not strongly reduced. Differences in intensity are, however, apparent upon software-based anaylses (Fig. 6). Images were generated with a Zeiss LSM880 confocal microscope. The excitation (ex) and emission (em) wavelengths are indicated in the images. Those shown in A-A″ were obtained by the normal confocal mode, while those shown in B-D were produced by the fast airyscan mode. Details of the respective settings are described in the Methods section. Labelling: co … coxa, fr … femur, tb … tibia, th … trochanter. The asterisk (*) marks auto-fluorescence of internal tissues after dissection. [file 12915_2020_902_MOESM11_ESM.jpg]

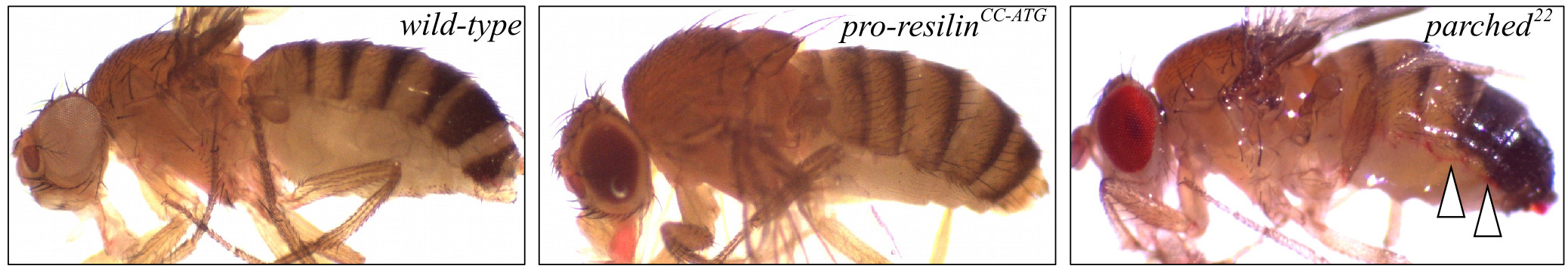

Supplement: Supplementary file 21 — Additional file 12: Figure S12. The tracheal systems of wild-type and resilincc-ATG flies are unstained after incubation with Eosin Y (red) that, by contrast, penetrates the tracheal system of parched22 flies (triangles), which have been described to have open spiracles [39]. Images were generated with a Leica EZ4HD with an in-built camera. [file 12915_2020_902_MOESM12_ESM.jpg]

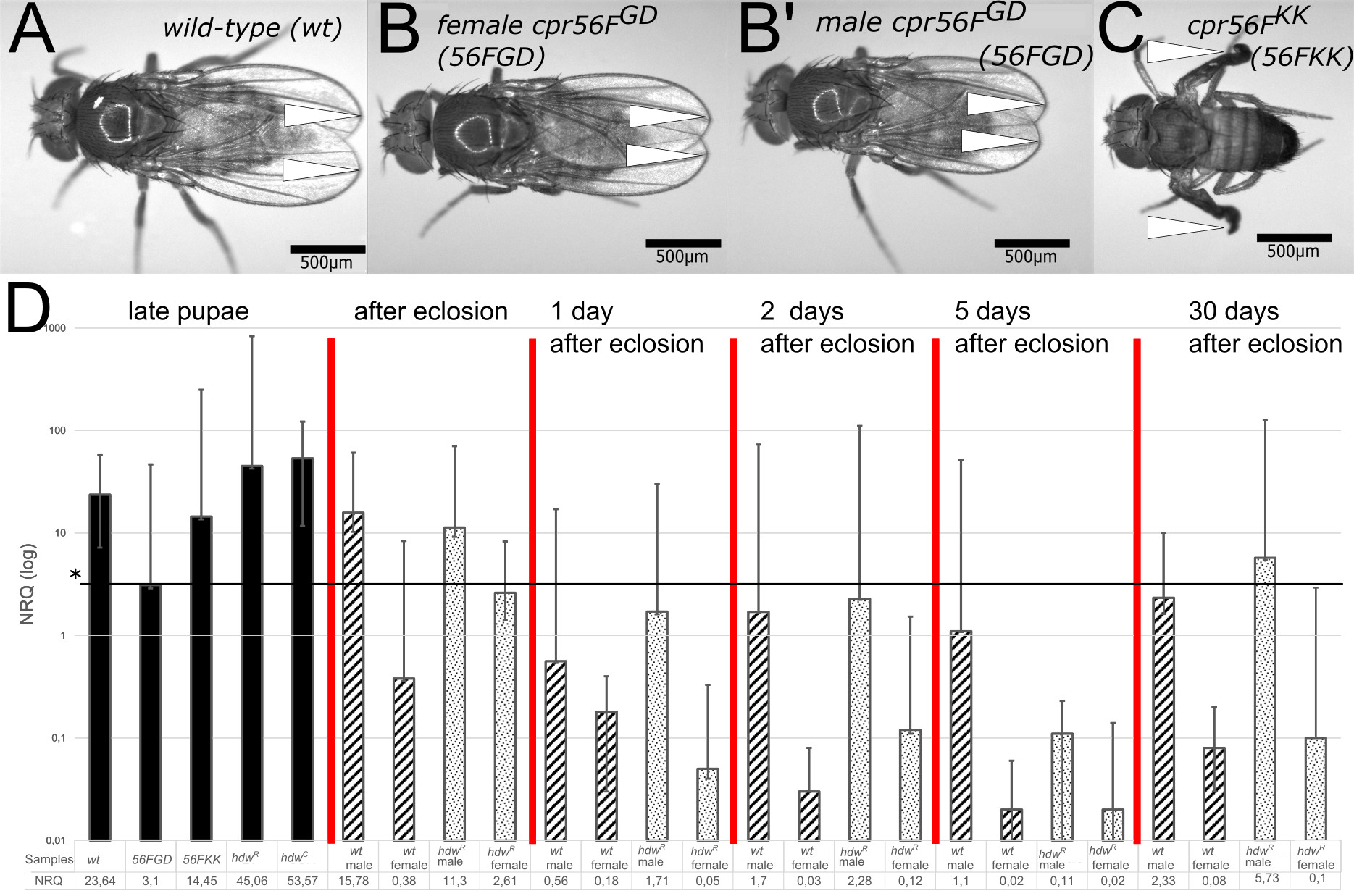

Supplement: Supplementary file 22 — Additional file 13: Figure S13. Reduction of Cpr56F does not cause any visible phenotype. (A) When resting, the wild-type fly holds its wings at the back. (B, B′) RNAi against cpr56F does not have any effect on wing posture. (C) Some flies with reduced cpr56F expression have crippled wings. (D) cpr56F is highly expressed in pupae. Its expression drops in eclosed flies. cpr56F expression is not strongly reduced by RNAi. According to the overlapping confidence intervals (CI), the differences in expression levels are not highly significant. Images were generated with a Leica EZ4HD with an in-built camera. [file 12915_2020_902_MOESM13_ESM.jpg]

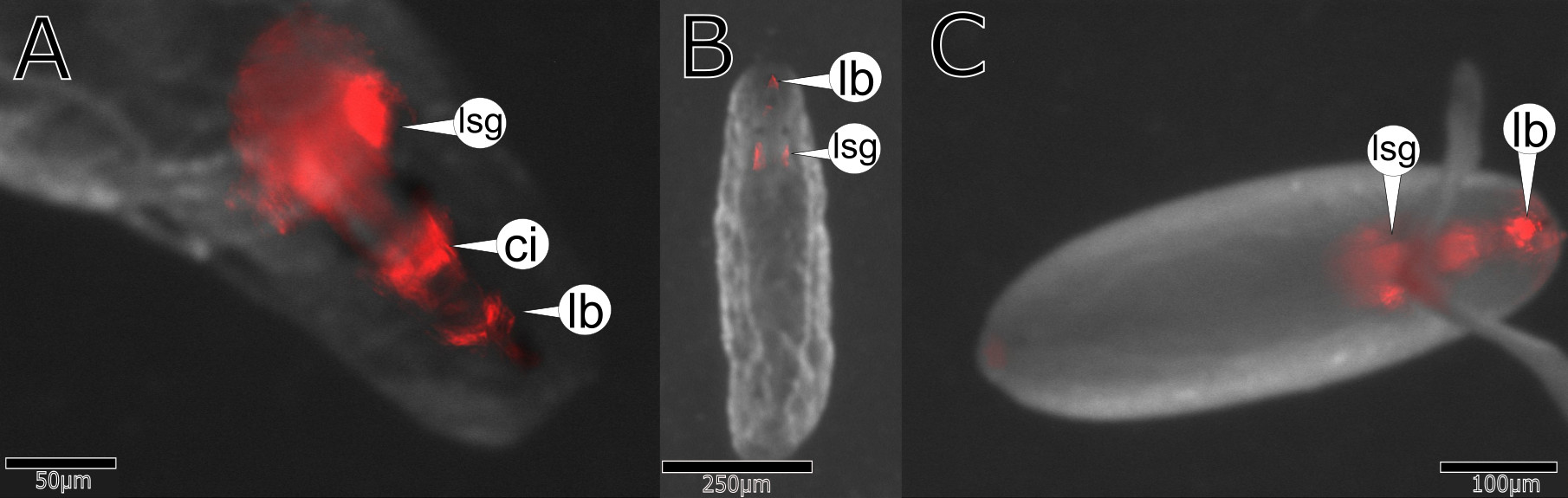

Supplement: Supplementary file 23 — Additional file 14: Figure S14. Cpr56F-GFP is detected in the head region of the embryo and larva. (A) The head skeleton and the tip of the head of the ready-to-hatch embryo contain Cpr56F-GFP. (B) This signal persists during larval stages. Images were generated with a Leica M205FA fluorescence binocular. Details of the respective settings are described in the Methods section. Labelling: lsg... larval salivary gland, ci...(larval) cibarium, lb... (larval) labellum. [file 12915_2020_902_MOESM14_ESM.jpg]

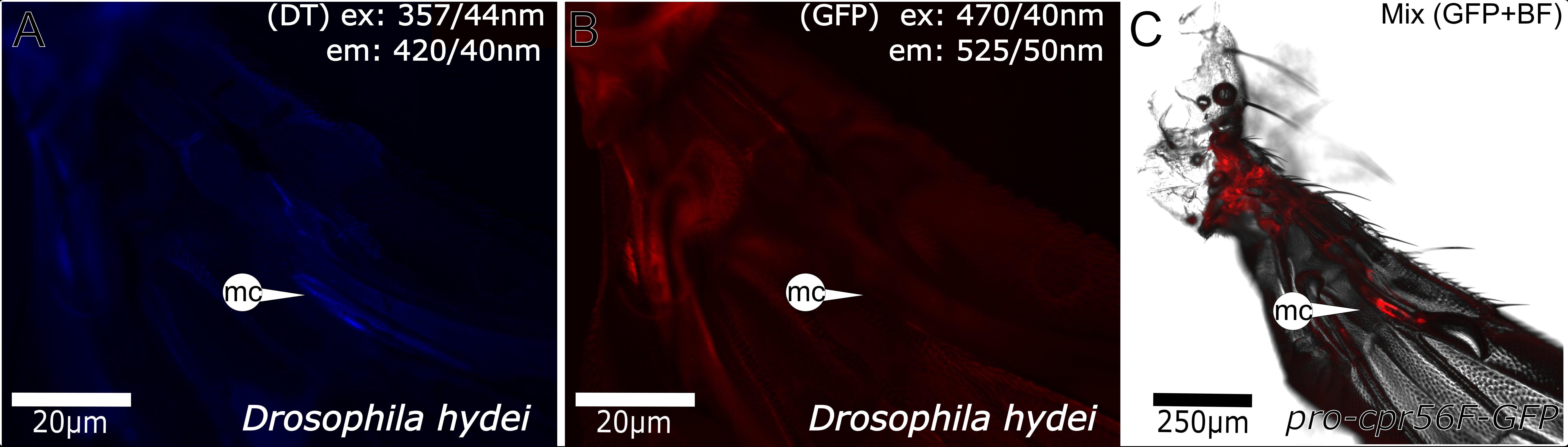

Supplement: Supplementary file 24 — Additional file 15: Figure S15. The proximal edge of the marginal cell (mc) in the wing of Drosophila hydei contains dityrosine (A). In the green channel, this signal is missing (B). Cpr56F-GFP is expressed at this position (C), whereas Pro-Resilin-GFP is not (see Fig. 1). The region of dityrosine and Cpr56F-GFP signal in the wing was named according to a reproduction of the wing at flybase (http://flybase.org/reports/FBim0000833). Here, we used an Axio Observer Z1 microscope for imaging. The excitation (ex) and emission (em) wavelengths are indicated in the images. Details of the respective settings are described in the Methods section. [file 12915_2020_902_MOESM15_ESM.jpg]
