# Supplementary material for: Resilin matrix distribution, variability and function in Drosophila
Source: BMC Biol. 2020 Dec 14;18:195. doi: 10.1186/s12915-020-00902-4 (PMC7737337; doi:10.1186/s12915-020-00902-4)
Supplement: Supplementary file 19 — Additional file 10: Figure S10. (A) The pro-resilin locus, genomic organisation and mutations. The two bases CA (green box) in the first exon (magenta letters) are deleted in the pro-resilinCC-ATG allele. This causes a frameshift and a premature stop codon resulting in a truncated protein as shown in Additional file 8: Figure S8. The three bases GCG (blue box) in the second exon (magenta letters) are deleted in the pro-resilinCC-RR allele. By this deletion that affects two consecutive codons, the amino acid M365 is changed to an I and the amino acid R366 is deleted as shown in Additional file 8: Figure S8. (B) The cpr56F locus, genomic organisation and mutations. The nine bases CGTGTCCCT (underlined green box) in the second exon (magenta letters) are deleted in the cpr56F12 allele. This causes a deletion of the three amino acids LVC in the signal peptide of the resulting protein as shown in Additional file 9: Figure S9. The cytosine2458 (framed box) in the second exon (magenta letters) is deleted in the cpr56F2 allele. This deletion causes a frameshift resulting in an aberrant protein sequence of 78 amino acids after L10 and a premature stop codon as shown in Additional file 9: Figure S9. [file 12915_2020_902_MOESM10_ESM.docx]

**Figure SG. The Crispr/Cas9 induced mutations in *pro-resilin* and *cpr56F*.**

(A) The *pro-resilin* locus, genomic organisation and mutations

GTCATAGCAACCACTGAACCACTGGAACACTGAACCACTCGCTTGGGAGAGTGAGCCGCG

GCAGACGCCGCCGACACATTCACCCAGTGGAGTAGACCGAGTAGACTACTTTGGGCCAAC

GCAACGGCGGATGCAAAAAGGTTTGGGGCCCGGGCACAATCACCACCTCGCCGCGCCTCG

AGAAGATGTGCACCGAAAGAAGGCAAATCAGCCCGAGACCCAAGACCGAGGCACCCTTTA

CGTGGCAATCTGGCGCAGATCGGGGTGGTGAAGTGTTGGCAAGCAAGCTCAGTGATTGAG

ATTTTTGCTTTCGACCGCATAGAATCAGCCACCGCTTCTCTGAAGACGATCGTATGTGGC

AGCCTCATCAGCTGCATCAGCAGCAACAGCAGCAGCAGCAGCTGTTGCGCTGGTTGCCAC

ACGCTCCACAAAACCGCCACCTCCAACTCATAGTCGAGTTCGTGTGTGTGCATCCTAGAC

TTTCAGTTTTTCGGTTTTGCCTCATATAAATACGCCATGTGGCAGCTACCACAGATCAGT

CAGAATTCGGCGCTGCTTCCAAGCGCTAGTCTCATCCCAAAGATTTCGATCAGTCCGTAG

ACCGAGCTTCAGTTCCAGCTTCAGCTTTAGATACAAGTGGAATATGTTCAAGTTACTCGG

CTTGACGCTGCTCATGGCAATGGTGGTCCTTGGGCGACCGGAGCCACCAGTTAACTCGTA

TCTACCTCCGTCCGATAGCTATGGAGCACCGGGTCAGAGTGGTCCCGGCGGCAGGCCGTC

GGATTCCTATGGAGCTCCTGGTGGTGGAAACGGTGGACGGCCCTCAGACAGCTATGGCGC

TCCAGGCCAGGGTCAAGGACAGGGACAAGGACAAGGTGGATATGCAGGCAAGCCCTCAGA

TACCTATGGAGCTCCTGGTGGTGGAAATGGCAACGGAGGTCGTCCATCGAGCAGCTATGG

CGCTCCTGGCGGTGGAAACGGTGGTCGTCCTTCGGATACCTACGGTGCTCCTGGTGGCGG

AAATGGTGGACGCCCATCGGACACTTATGGTGCTCCTGGTGGTGGTGGAAATGGCAACGG

CGGACGACCTTCAAGCAGCTATGGAGCTCCTGGTCAAGGACAAGGCAACGGAAATGGCGG

TCGCTCATCGAGCAGCTATGGTGCTCCTGGCGGTGGAAACGGCGGTCGTCCTTCGGATAC

CTACGGTGCTCCCGGTGGTGGAAACGGTGGTCGTCCTTCGGATACTTACGGCGCTCCTGG

TGGCGGCAATAATGGCGGTCGTCCCTCAAGCAGCTACGGCGCTCCTGGTGGTGGAAACGG

TGGTCGTCCATCTGACACCTATGGCGCTCCTGGTGGCGGTAACGGAAACGGCAGCGGTGG

TCGTCCTTCAAGCAGCTATGGAGCTCCTGGTCAGGGCCAAGGTGGATTTGGTGGTCGTCC

ATCGGACTCCTATGGTGCTCCTGGTCAGAACCAAAAACCATCAGATTCATATGGCGCCCC

TGGTAGCGGCAATGGCAACGGCGGACGTCCTTCGAGCAGCTATGGAGCTCCAGGCTCAGG

ACCTGGTGGCCGACCCTCCGACTCCTACGGACCCCCAGCTTCTGGATCGGGAGCAGGTGG

CGCTGGAGGCAGTGGACCCGGCGGCGCTGACTACGATAACGATgtaagtgtttgagatag

cttagcaaatagggtatatggtaaaatgggatttgtatacggggatcactgggtggaatt

gaccacaagggaacaccgctccaccgcccacaccgctgcacacttgcccttgcaaatctt

tggtagactcgttatcgctcacctgcacagccatcggcgtttaaagcttgcatccccata

gaaaactgaattcttgatcccagacactctatctctctgtgtttctctctttctccacac

gccccaatccgttctccattctccatgatccgctgtccatgatccatgctccatgctcca

tgatccgttgtacggcgtttagGAGCCCGCCAAGTACGAATTTAATTACCAGGTTGAGGA

CGCGCCCAGCGGACTCTCGTTCGGGCATTCAGAGATGCGCGACGGTGACTTCACCACCGG

CCAGTACAATGTCCTGTTGCCCGACGGAAGGAAGCAAgtaagcggcggcggcagcgatgg

cgggcagcagaaggcgatgtggatccacccactgctccaccacctcatccttctgtcacg

gctaacattgttatctcgttctcttttctctttgtctttcttagATTGTGGAGTATGAAG

CCGACCAGCAGGGCTACCGGCCACAGATCCGCTACGAAGGCGATGCCAACGATGGCAGTG

GTCCCAGCGGTCCTGGAGGTCCTGGCGGTCAGAATCTTGGTGCCGATGGCTACTCCAGTG

GACGTCCCGGCAATGGAAATGGCAACGGAAATGGCGGTTACTCCGGTGGACGTCCAGGAG

GCCAGGATTTGGGACCTAGTGGATATTCCGGTGGTCGTCCAGGAGGTCAGGATCTAGGCG

CCGGTGGCTACTCCAATGGCAAGCCGGGCGGCCAAGACTTGGGACCAGGCGGTTACTCCG

GTGGTCGCCCTGGAGGTCAGGACTTGGGTCGAGACGGCTACTCCGGTGGACGTCCAGGTG

GACAGGACCTCGGTGCCAGCGGCTACTCCAATGGTAGGCCAGGCGGCAATGGCAACGGTG

GATCCGATGGCGGTCGTGTGATCATCGGTGGACGTGTGATAGGCGGCCAGGATGGCGGTG

ATCAGGGCTACTCCGGCGGACGTCCCGGTGGTCAGGATCTTGGACGTGATGGCTACTCCA

GCGGTCGTCCTGGTGGTCGGCCAGGCGGCAACGGCCAGGATAGTCAGGATGGCCAAGGAT

ACTCGAGCGGCAGGCCGGGTCAGGGTGGCCGGAATGGATTCGGACCCGGTGGTCAGAACG

GTGACAACGATGGCAGCGGTTATCGGTACTAGGAACGACTCACAGACACATTTAGCTAGA

TTCCCCGAACATATAGCAATACCTAGTGTTAAGTCCTTCTAGAACTATGTCCCCTACCTA

TGCCCCTCATCCTTCCGCATCTATTCGTGCCCATTCTATCCCACTTCTTGCTGTCTTTCG

CCCATCCATTCAGCCTGCTCAATTCCTCGTAGTCGTAAGTCCCTGTTGATCTAAATGCAA

GTGCGAAAAATAACCCAAAAAAAGGAATGAAATGATAAAGCTACTTCCGATGTAAGTAAA

GAAATTAAATACATATATATTTTTTAT

The two bases CA (green box) in the first exon (magenta letters) are deleted in the *pro-resilin^CC-ATG^* allele. This causes a frameshift and a premature stop codon resulting in a truncated protein as shown in figure S4.

The three bases GCG (blue box) in the second exon (magenta letters) are deleted in the *pro-resilin^CC-RR^* allele. By this deletion that affects two consecutive codons, the amino acid M^365^ is changed to an I and the amino acid R^366^ is deleted as shown in figure S4.

(B) *Cpr56F* locus, genomic organisation and mutations

TCAGTATCACAATCAAGTCAACCATCTTCGCAAGAAGAACACAGCCAACAGAGCAAAGTT

AACAAAAACCGTTTTCGTGTGTGCCAAAATCCCAAACAAGTGACAACACAAAATGAAGgt

atatagccgaatagaactttcgagtcaagtgccaagtggtgtgctgtgtgaaaaaaaaaa

gtcctcacaaaaggatatgggcagccaaaacgaaatacaatgcctcattcggcattcggg

aaacgatatgactaaaggatataaaacttgaatagctataaagatcattcaatataggtc

ctttattagatacagtttcgaccttttgttaaattttcaaaggatattgaagtttgctgt

agctaacatacaaacaacgttgggttacaaaatcttagaatgatcaaataaacatcacaa

tatttgcccatatctttttctgcattgcaaaggtgctaaaatatgttcgaattttgtatt

catttttctgtatccaatcaatagccctcatttaaaatcgtttttggcaatagatcgatc

aatcaattaaacaactcatcaaactaatgaagctttcattgtctcacatagcacttaatc

agctaatcggaaggaaaagacatgaaatattaatatttgagccatattaaaagtcaagtt

cagctgggtgattaatcaagcggagatccgcataataaatttcataagttgccatcatca

tcgcagttaagattaccatcaatttgtttaggatagctactgttttttatggtaggttgc

ggaattagaacaccgatatcattcgcctgggaaacaattgcaaccagcgatcccagttca

tttctagggtgtgggtaacaaacagaagcaacaacagctgccgctgcttggggtcatttt

gcaatgaataaattagagaaactttaagtggcggaagaagaactactggacatgcccaag

taaggcagtctggagatgaccaggcggcgataaaaaaaaaatacagacaaaagcgcagcc

aaaaagcatagcattttaacgccaatgaagcatgaaagtcattgacggccctgtgcctaa

acactcggccagaagatcttttgcacaagattcggaaataagttgtgtaagagtgagccg

aagtggttctcccacaggttgtccgcgccacacggcgaatacttaagattcagacgcatt

gcgcaagatcacgagattagcccttcacttgcagcccagctaaatccaatccaatccgat

gggagcttcacctgggacccaattactacagatttgcatatgcctcagctaaatgctgta

aagatgggaaagacaaagccaattctgggcctgctcgcaggccatcaaatccaaacaaaa

gattgtcaagcgggtCACAGCCTGAGCAGTTATTGTTATGAGAGTTTAATGAGCTGGCAG

CCAGCCGGGCGAACAGTGCCCACTTATTCGAGAAGTACGCGACAGTTGCTTATGAGAAAT

TTTAGCTTTAGGATCAAATGTGGGATGGGCTATCGATTTCTCCTACTGGCGTCATTGTCA

CATCTTTTTGGATCTCAGCGGCAGTACGGTAGGCCGTGGGGTGGATTTTAAAGCCATAAA

GCctgggaaaacatagttacattagttatacattattagtaactaccttaaaaacatagg

gaaatagaaggaaacacaggaaacggaagatctaacCTGGGCACAAACTTCTGCTTTGCC

CGCTTGGGCCTAGCATCCGGTTTCTCCATAAAGAACATATGAGGGAATCCAGTGCCAAAG

AAGGCTCCATCCAGATTCGAATGTCTCGACGCCTTGGGAATGTACACATCATTGCACTTG

GGGCAATAGATGCGTACCATATCCTCGCCAGGATTATCACTCAAACCAATGGGCAGCACC

GGCTGGCTATGGCAGAATGCACGTGGACATGTGCCGAATTCGCCCTTGTTGTACTTGTCC

AACATCAGTTCGATGCCCCTGTTGGTTAGGATAAAGCGAGCATGGATCAAGCCATACAGT

TTCTCTGCACTAGCCTCCAGTTCTGGTTCAGCGGGGTCTTCCGAAGCTGAACCCGGATTA

AGATCCAGGATCACCTCCAAGGCACATTTGTAGTTCTTAACGTTCGAATCAAGAAAGTTA

AGGTTAAACTTGTCCTGGATATATTCCTCATCCACCTCGCAAAAGAATTCGTTGCCACGC

TGCTTGCAAAACCAATGAATCCAGGAAGATTCGTCCGAATCGGTCATAGCTGCTTCTTTT

AAGCTTTAATTTAATCAGTCAAAATCACGAGAGCTTAACAATAGGACTTATTTAATTTCG

AATGACAGCTGTCAGGGACGGAGTGTTCAATCAAGGGTTGTCAACTCAAAACGAAAACTG

TaatgtaaatgcaaccctgaattttcattcccagGCTTTTACATCCATTGCCCTGCTCGT

GTGCCTGGCTGCCTGGACCCATGCGGAACCACCAGTTCCCCAGAACCAGTACCTGCCCCC

CAATCAGTCGCCTCAGGCGCCGTCCAACAACTACCTGCCGCCCACGCAGGGCTACCAGTC

GCCGTCGAGCAACTACTTGCCACCCCAGCGGGCCGGTGGCAATGGAGGAGCGCCCAGCAA

CAGCTATGGCGCCCCCATCGCTCCTCCCCAGGGTCAATATGGTGCTCCAGCGCTGACTGG

TGCCATCTTCAAGGGCGGAAACGGAAACGGAAACGGCGGCTATGGCGGCGGTAATGGCAA

CGGCAACGGCTACGGACAACGGGATGAGGAGCAGTATGGACCGGCCAAGTACGAGTTCAA

GTACGACGTACAGGACTACGAGTCGGGCAATGACTTCGGCCACATGGAGTCCCGCGATGG

TGATCTTGCTGTGGGCCGCTACTACGTCCTGCTGCCCGATGGACGCAAACAGATTGTTGA

GTACGAGGCCGACCAGAATGGATACCGCCCAACCATTCGGTACGAGCAGGTCGGCAATGG

CAACGGAAACGGCAATGGCAATGGTCGCAACGGTGGCGGTTATGATAGCAACGCGCAGCA

GGGCAAATTCAACGGCTACTAAGATGGGGAAGTGAGGATACCCGATTTAGGTCGACCCCG

CAACTATACTCGTCTACTTCATGGGATCGCTGGCCGAGTATTAAACGCACATCCAGCCAG

TGGGCCAGCGATCGCAGTCAGTCTATCCATATAACCCTATTCCCATCTCTGTGCATACCC

TTAGTGGGACGCCCGCCATTTTGCCATAGACAGGCGCGTTCCTTTCCGTTTCCGTTTCAC

TGCTGTTCGTTATCCTGTTTTCCGTCTCTACACCTGGAACTCCTTGCCGTTTTGTTTTGT

TTAAGCGCCTTCATTTTGTGTTAGTCTACGCACACACTCCAACACACCATTCACCCACAA

CATGACCATTACGCATTCATACATACATGCATAAGGATTGTATTTAGTTTTTAAACGAAA

ATGGAAATTCGAAAGTGAAACGAACTTATGAGATTTTTGTGTATATTTAATAATTATTAA

ATGAATAAAGAAGCATTTTTGTATAAAACAAAGTC

The nine bases CGTGTCCCT (underlined green box) in the second exon (magenta letters) are deleted in the *cpr56F^12^* allele. This causes a deletion of the three amino acids LVC in the signal peptide of the resulting protein as shown in figure S4.

The cytosine^2458^ (framed box) in the second exon (magenta letters) is deleted in the *cpr56F^9^* allele. This deletion causes a frameshift resulting in an aberrant protein sequence of 78 amino acids after L^10^ and a premature stop codon as shown in figure S4.
