# Supplementary material for: Resilin matrix distribution, variability and function in Drosophila
Source: BMC Biol. 2020 Dec 14;18:195. doi: 10.1186/s12915-020-00902-4 (PMC7737337; doi:10.1186/s12915-020-00902-4)
Supplement: Supplementary file 25 — Additional file 25: Text. Extended description and discussion of Resilin patches in the fly leg. [file 12915_2020_902_MOESM25_ESM.docx]

**Additional file s25
Resilin patches in the *D. melanogaster* leg**

**(specified as l + number in Additional file 3: Figure S3A–C)**

**l1**: A patch at the dorsal articulation of the coxa. It may reflect high resilin content or strong thickening of the cuticle. There is no patch evident representing any other articulation of the coxa.

**l2**: A patch at the ventral border between coxa and trochanter, possibly upon the basal rim of the trochanter, or shortly proximad of it. This could be the widened subbasal part of the tendon bearing the attachment of the trochanteral depressor muscle (Soler et al. 2004: tendon trdt and muscle trdm in figure 6B; therein trdt is shown as widened subbasally).

**l3**: A small patch at the dorsal border between coxa and trochanter, likely upon the basal rim of the trochanter. This could be the origin of the tendon bearing the attachment of the trochanteral levator muscle (Soler et al. 2004: tendon trlt and muscle trlm in figure 6B) or perhaps the nearly tendonless attachment area of the trochanteral rotator muscle (Soler et al. 2004: muscle trrm in figure 6B).

**l4**: A small patch dorsallyon the trochanter; it could be part of **l5**, though it appears to be a separate patch.

**l5**: An apparently circumferential ring at the distal part of the trochanter. This suggests that the border between the trochanter and the femur, along which these two podomere sclerites are more or less fused, is still functionally relevant in the fly. This is also suggested by the presence of a trochanterofemoral muscle (Soler et al. 2004: fedm in figure 6B). The stained ring may well include basal parts of the tendons arising at the femur base and bearing the trochanterofemoral muscles (Soler et al. 2004: tendons fedt, fert and muscles fedm, ferm in figure 6B). However, due to its orientation, it appears unlikely that patch **l5** only represents parts of these tendons.

**l6**: A patch at the dorsal border between femur and tibia, likely upon the basal rim of the tibia (countersunk into the femur). This likely represents the widened origin of the tendon bearing the attachment of the tibia levator muscle (Soler et al. 2004: tendon tilt and muscle tilm in figure 6B; Snodgrass 1935: muscle lv in figure 105C).

**l7**: A patch at the ventral border between femur and tibia, likely upon the basal rim of the tibia. This likely represents the widened origin of the tendon bearing the attachment of the tibia depressor muscle (Soler et al. 2004: tendon tidt and muscle tidm in figure 6B; Snodgrass 1935: muscle dpr in figure 105C).

**l8**: A patch at the side-part of the border between femur and tibia (possibly anteriorly and posteriorly, covering each other). This likely represents the anterior and/or posterior articulation between femur and tibia; staining may be due either to high Resilin content or to cuticular thickening (Snodgrass 1935: articulation at l in figure 105B, C).

**l9**: A large, structured patch at the border area between the tibia and the first tarsomere (detailed in figure S3B); it cannot be exactly located, but rather seems to concern the tarsomere base. This may represent the widened origins of the tendons bearing the attachments of the tarsal levator and depressor muscles (Soler et al. 2004: tendons talt, tadt and muscles talm, tadm in figure 6B). They are likely seen in an aspect where they cover each other, whereby a differentiation between them is not possible in the picture. In addition, tibiotarsal articulations may be included in the patch as well.

**l10**: Sporadic (Figure S3A) or nearly continuous (Figure S3C) streaks in all tarsomeres. This might be parts of the long tendon arising from the unguitractor plate on the ventral side of the pretarsus (Soler et al. 2004: tendon lt in figure 6B; for depressing the claws). It is obscure why staining is stronger in selected parts throughout the tarsus and does not extend upward inside the tibia (as the tendon does).

**Conclusions:** Part of the staining pattern clearly corresponds with the basal portions of the tendons in the leg (l2, l3, l6, l7, and perhaps l4, l9, and l10). All known tendons of the leg are represented (at least potentially if resolution is low). Staining of tendon bases is quite certainly due to high Resilin content. Other parts of the staining pattern correspond with articulations (l1, l8, l9). Again, all known articulations of the leg are potentially represented, with the exception of the ventral coxal articulation. Staining of articulations may be due either to high Resilin content or to cuticular thickening. The distitrochanteral ring is a special case.

**Literature**

Snodgrass R. E .(1935). Principles of Insect Morphology. New York, NY: McGraw-Hill.

Soler C., Daczewska M., Da Ponte J.P., Dastugue B. and Jagla K. 2004: Coordinated development of muscles and tendons of the *Drosophila* leg. Development 131 (24): 6041-6051. doi:10.1242/dev.01527
